# TAMOF-1 for capture and separation of the main flue gas components†

**DOI:** 10.1039/d5ta01362c

**Published:** 2025-04-29

**Authors:** S. Gooijer, S. Capelo-Avilés, S. Sharma, S. Giancola, J. R. Galán-Mascaros, T. J. H. Vlugt, D. Dubbeldam, J. M. Vicent-Luna, S. Calero

**Affiliations:** a Materials Simulation and Modelling, Department of Applied Physics and Science Education, Eindhoven University of Technology PO Box 513 5600MB Eindhoven The Netherlands j.vicent.luna@tue.nl s.calero@tue.nl; b Institute of Chemical Research of Catalonia (ICIQ-CERCA), The Barcelona Institute of Science and Technology (BIST) Av. Països Catalans 16 Tarragona 43007 Spain; c Engineering Thermodynamics, Process & Energy Department, Faculty of Mechanical Engineering, Delft University of Technology Leeghwaterstraat 39 2628 CB Delft The Netherlands; d Orchestra Scientific SL Av. Països Catalans 16 Tarragona 43007 Spain; e Catalan Institution for Research and Advanced Studies (ICREA) Passeig Lluís Companys 16 Barcelona 08007 Spain; f Van't Hoff Institute for Molecular Sciences, University of Amsterdam Amsterdam Netherlands

## Abstract

Experimental screening of Metal Organic Frameworks (MOFs) for separation applications can be costly and time-consuming. Computational methods can provide many benefits in this process, as expensive compounds and a wide range of operating conditions can be tested while crucial mechanistic insights are gained. TAMOF-1, a recently developed MOF, stands out for its exceptional stability, robustness and cost-effective synthesis. Its good CO_2_ uptake capacity makes it a promising agent for flue gas separation applications. In this work, we combine experiments with simulations at the atomistic and numerical level to investigate the adsorption and separation of CO_2_ and N_2_. Using Monte Carlo simulations, we accurately reproduce experimental adsorption isotherms and elucidate the adsorption mechanisms. TAMOF-1 effectively separates CO_2_ from N_2_ because of preferential binding sites near Cu^2+^ atoms. To assess separation performance in equilibrium at different conditions along the entire isotherm pressure range, adsorbed mole fractions, selectivities, and the trade-off between selectivity and uptake (TSN) are calculated. The dynamic separation performance is assessed by breakthrough experiments and numerical simulations, demonstrating efficient dynamic separation of CO_2_ and N_2_, with CO_2_ being retained in the column.

## Abstract 2

Atomistic and numerical simulations are combined with experiments to investigate the adsorption and separation performance of TAMOF-1. It was found that TAMOF-1 can efficiently separate CO_2_ from N_2_, which is relevant for flue gas applications.

## Graphical abstract

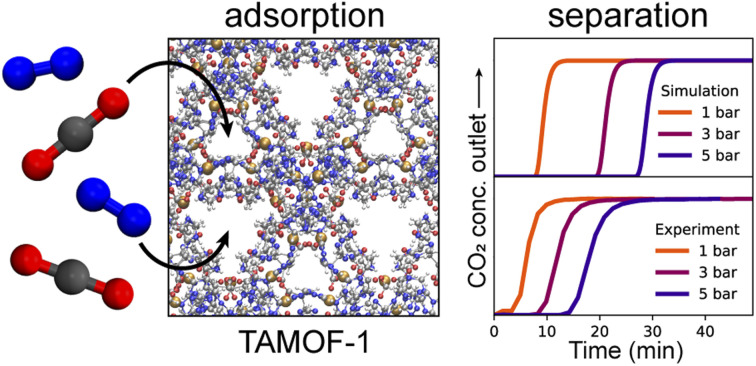

## 1 Introduction

Metal Organic Frameworks (MOFs) are a class of porous crystalline materials that consist of inorganic metal cluster nodes connected to organic linkers. The metal and organic building blocks can be varied enormously, leading to MOFs having large chemical and structural versatility. MOFs can be synthesized in a modular fashion, where the building blocks self-assemble into a predetermined crystal structure.^1^ The resulting crystals often have high surface areas, porosities, and thermal stabilities, making them attractive agents for adsorption-based separation applications.^2–12^

Separation of gas mixtures can be achieved using MOFs because of preferential adsorption of a certain component, which occurs for several reasons. In certain instances, this is caused by shape and size effects of the adsorbates if certain components cannot enter the framework or diffuse at a much lower rate.^13^ There can also be specific adsorbate–surface interactions at play that make it energetically favorable for a component to adsorb onto the surface. Whether a MOF is suitable for a specific separation application depends on many factors, as the types of adsorbates, mixture composition, and operating conditions affect the separation performance. In general, the desired MOF should exhibit high adsorption capacity and selectivity for the target compound, along with low regeneration costs. Additionally, its synthesis should prioritize the use of earth-abundant metals and scalable production methods.^14,15^

Simulations on the atomic scale based on a theoretical model can provide many benefits in the screening of MOFs for separation applications, as experiments can be costly and time-consuming. Many adsorbates can be easily investigated, including compounds that might be toxic or rare. A wide range of operating conditions can also be tested, so the performance of materials can be optimized for different applications. Additionally, atomistic simulations provide information on the microscopic interactions between the guest molecule and the framework. These interactions often drive the adsorption, so this detailed understanding aids the design and fine-tuning of MOFs. Many computational studies of adsorption in MOFs have been carried out, for instance to screen MOFs for CO_2_ adsorption and separation.^16–19^

Energy efficient removal of CO_2_ from various gas mixtures is a widely researched application aimed at mitigating climate change. Separation of flue gas is especially important, as flue gases emitted from, for instance, fossil burning power plants are point sources responsible for large CO_2_-emissions. Flue gas contains around 10–15% of CO_2_ and more than 75% of N_2_.^20,21^ The low concentrations of CO_2_ in flue gas make CO_2_ separation challenging and energy intensive, highlighting the need for efficient and cost-effective CO_2_/N_2_ separation technologies. Currently, the commercially available CO_2_-capturing technologies such as amine scrubbing are accompanied by high energy consumption.^22,23^ Adsorption of CO_2_ in MOFs can be much more energy efficient, as adsorption of CO_2_ in MOFs is not of a chemical but physical nature, which makes regeneration of the material much easier.^24–30^

In this work, we will explore the Triazole Acid Metal Organic Framework (TAMOF-1), which can be easily synthesized by adding a l-histidine derivative to a copper(ii)-salt in water, reagents which are cheap and easily accessible.^31^ The resulting framework is highly thermally and chemically stable and can withstand polar and apolar solvents, as well as complete solvent loss. As the ligands are enantiopure, the resulting framework is homochiral, making it possible to perform enantioselective separations in the liquid phase.^31,32^ Separations of gas mixtures of achiral organic compounds have also been carried out with TAMOF-1, which proved to be possible due to the distinct shape and size of the pore network of TAMOF-1.^33^ Recently, the ability of TAMOF-1 to capture CO_2_ from a methane based gas mixture for biogas upgrading was also explored, which showed excellent selectivity and good CO_2_ uptake.^34^ However, no extensive analysis on the separation performance was performed. With the good CO_2_-uptake, TAMOF-1 also seems suitable for flue gas separation applications, but the low concentration of CO_2_ in flue gas may pose a challenge. This low concentration translates to low partial pressures, which can reduce adsorption capacity if the MOF exhibits high saturation loading but weak CO_2_ binding at low pressures. Additionally, compared to CO_2_/CH_4_ separation, the MOF requires different regeneration conditions, which may be less energy-efficient for flue gas applications. These factors make it crucial to perform a systematic and extensive evaluation of the separation performance of this promising adsorbent at different conditions to efficiently recover CO_2_ from flue gas.

The goal of this work is to combine experiments with both atomistic and numerical simulations to evaluate the adsorption and separation performance of TAMOF-1. First, the framework of TAMOF-1 will be characterized with computational methods that will provide crucial insights into adsorption mechanisms of different adsorbates. As TAMOF-1 is a promising agent for the separation of CO_2_ from flue gas, mixtures of CO_2_ and N_2_ will be chosen as the gas system to demonstrate our model. The pure component isotherms of CO_2_ and N_2_ will be simulated and the adsorption mechanisms and properties of both compounds are determined. Next, equilibrium adsorption from mixtures of CO_2_ and N_2_ will be calculated, as well as dynamic adsorption in the form of breakthrough curve simulations. To assess the separation performance of TAMOF-1, adsorbed mole fractions, selectivities, and the trade-off between selectivity and uptake (TSN) will be calculated, which together can provide a comprehensive picture of the predicted separation performances at different conditions. The simulation results will be compared to the experimental data to validate the performance of the theoretical model.

## 2 Methodology

### 2.1 Experimental details

TAMOF-1 ([Cu(S–TA)_2_]·*x*H_2_O, *S*-HTA = (*S*)-3-(1*H*-imidazol-5-yl)-2-(4*H*-1,2,4-triazol-4-yl)-propanoic acid) was prepared in powdered form following the procedure as described by Corella-Ochoa *et al.*^31^ Single-component adsorption isotherms for CO_2_ and N_2_ were measured at a temperature range of 293–353 K up to a pressure of 10 bar. The detailed adsorption isotherm measurement procedure is described in Section S2.1.†

Breakthrough measurements were conducted at three gas flow rates: 86, 177, and 269 mL min^−1^, corresponding to gas velocities of 0.005, 0.01, and 0.015 m s^−1^ respectively. Each flow rate was tested across temperatures of 25–80 °C and pressures of 1–5 bar. The effect of the CO_2_ concentration (1%, 2.5%, and 6% in He) was specifically assessed at 25 °C and 1 bar using a gas velocity of 0.005 m s^−1^. Between measurements, the column was purged at 80 °C. First, N_2_ (100 mL min^−1^) flowed for two hours to remove residual gases. Then, helium (30 mL min^−1^) flowed until CO_2_ was no longer detected and the N_2_ baseline was re-established. The concentration at the gas outlet was continuously monitored until equilibrium was reached (*C*/*C*_0_ = 1). The dead volume of the gas in the setup was evaluated and subtracted for each operating condition. Detailed description of the experimental set-up is provided in Section S4.1.†

### 2.2 Simulation details

For the characterization of the TAMOF-1 framework, the helium void fraction, the pore volume, and the pore size distribution were calculated using the RASPA software.^35^ The theoretical X-Ray Diffraction (XRD) pattern was determined from the coordinates of the framework using Mercury.^36^ To visualize the accessible pore volume, PoreBlazer was used with a probe diameter of 3 Å.^37^

To predict adsorption isotherms, we performed classical grand-canonical Monte Carlo simulations using the RASPA software.^35^ After 10 000 initialization cycles, a production run of 50 000 cycles was run and the average number of adsorbate molecules present in the framework during the simulation was taken as the loading. During each cycle, *N* trial moves were performed, with *N* being the number of molecules. In conjunction with the loading, the isosteric heat of adsorption was computed using a fluctuation formula.^38^ Both the TAMOF-1 framework and the adsorbates were kept rigid during the simulations. van der Waals and electrostatic interactions were described by Lennard-Jones and coulombic potentials, respectively.

The Lennard-Jones parameters for the TAMOF-1 atoms were taken from the DREIDING force field, except for the copper atoms, for which the UFF was used (Table S1†).^39,40^ The charges for the framework atoms were taken from a previous work of TAMOF-1 which were calculated using the charge equilibration method implemented in RASPA (Table S1†).^31^ Previously reported models were used for CO_2_ (ref. 41) and N_2_ (ref. 42) (Table S2†). Shifted Lennard-Jones potentials with a cut-off of 12 Å without tail-corrections were used and Lorentz–Berthelot mixing rules were applied to calculate the cross-interaction terms.^43,44^ The Ewald summation method was used for the evaluation of the electrostatics.^45^

The adsorption energy and configuration of CO_2_ were calculated by performing periodic DFT-calculations. The calculations were performed using the Quickstep module of the CP2K-program.^46^ The Perdew–Burke–Ernzerhof (PBE) functional along with Grimme's dispersion corrections (D3) was used.^47–49^ Double-*ζ* atomic basis sets were used along with an auxiliary plane wave basis set with a cutoff energy of 750 Ry.^50^ Detailed input parameters can be found in Section S2.3.†

The prediction of adsorption from mixtures was performed with the RUPTURA software, predicting both equilibrium mixture adsorption and breakthrough curves.^51^ To predict the adsorption from mixtures, the pure component adsorption isotherms from the Monte Carlo simulations were first fitted to the multi-site SIPS equation to achieve the best fit of the data (eqn (1)).^52^1
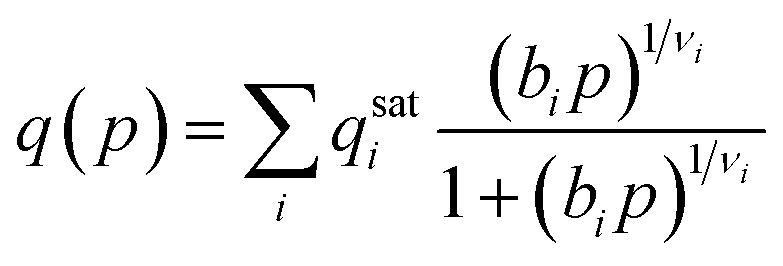
Here *q*(*p*) is the absolute loading of the adsorbed phase, *q*^sat^_*i*_ the saturation loading, *p* the pressure and *b*_*i*_ and *ν*_*i*_ the affinity and Henry coefficients, respectively. The predictions of multicomponent equilibria isotherms and breakthrough curves were then performed using Ideal Adsorption Solution Theory (IAST).^53^ Detailed run settings and column parameters can be found in the Section S4.†

To test the influence of the temperature on breakthrough times of CO_2_, isotherms of additional temperatures were generated. This was not done by running Monte Carlo simulations, but rather with a mathematical model based on the adsorption potential theory, a theory first derived by Polanyi and later refined by Dubinin.^54–56^ According to this theory, adsorption occurs due to an adsorption potential created by the adsorbent surface that depends on the distance between the adsorbate and the surface. The relation between the adsorption potential and the filled adsorption space is reflected in a characteristic curve, which is unique for each adsorbate–adsorbent pair and does not depend on the temperature. Recently, a mathematical model was developed that uses this characteristic curve to predict several isotherms at different temperatures from a single isotherm nearly instantaneously.^57^ Using this model, the isotherms of a range of adsorbates, including CO_2_, could be successfully predicted in several MOFs.^57^ More details of the model can be found in Section S2.5.†

Several metrics were used to properly assess the separation performance of TAMOF-1. Along with the absolute adsorbed amount, the adsorbed mole fraction of each component was computed, which is the productivity if we assume complete regeneration of the material. The selectivity, as given by eqn (2), was also determined.2
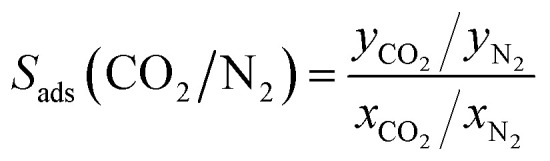
Here *S*_ads_(CO_2_/N_2_) is the selectivity of adsorption of CO_2_ over N_2_, *y* is the mole fraction of the adsorbed phase, and *x* the mole fraction of the bulk phase of CO_2_ and N_2_. For optimal CO_2_/N_2_ separation performance, a material ideally possesses a high selectivity with an absolute CO_2_-uptake still sufficiently high. For most materials, there exists a trade-off between these two properties.^58^ The metric that quantifies this for different separations is the trade-off between selectivity and uptake (TSN) as given by eqn (3).^59,60^

3 TSN_(CO_2_/N_2_)_ = ln(*S*_(CO_2_/N_2_)_)*q*_CO_2__

## 3 Results and discussion

### 3.1 Characterization TAMOF-1

**Fig. 1:**
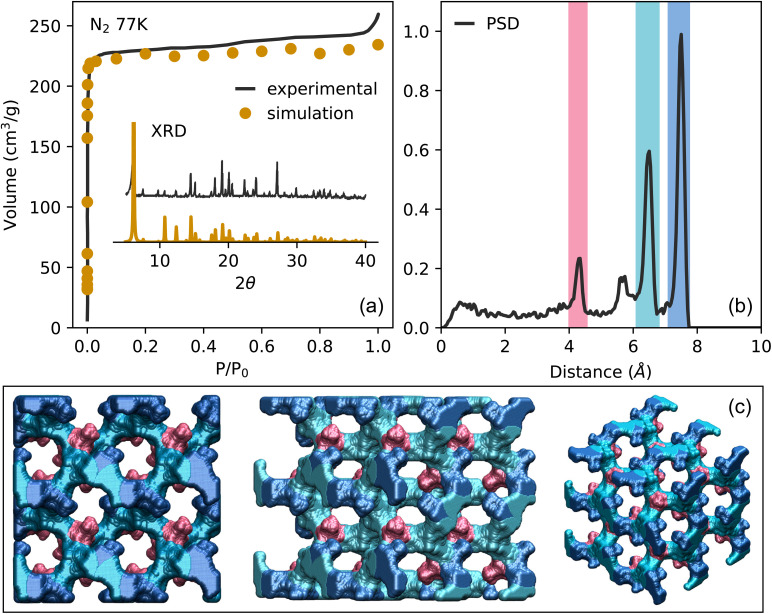
(a) Experimental and simulated adsorption isotherm of N_2_ in TAMOF-1 at 77 K (main) along with the XRD-spectra (inset) *P*_0_ is the saturation pressure of N_2_ at 77 K (1 bar). (b) Pore Size Distribution of TAMOF-1 simulated with RASPA.^35^ (c) Three different views of the accessible pore volume of TAMOF-1. The different regions of the void space correspond to the three prominent peaks of the PSD, as indicated by the color. The framework atoms are omitted here for clarity, but are shown along with the void spaces in Fig. S1.†

To accurately predict and understand the adsorption processes in TAMOF-1, the crystal structure needs to be elucidated along with its porosity properties. The BET surface area and the pore volume have previously been experimentally determined by measuring the N_2_ adsorption isotherms at 77 K.^31^ This experimental isotherm is in close agreement with our simulations, with a maximum deviation of 10 cm^3^ g^−1^, demonstrated in Fig. 1a. Table 1 compares the resulting surface areas and pore volumes between the experiments and our simulations. Again, these values are in very close agreement. The derived crystal structure used in the simulations can therefore be assumed to be close to experimental reality. This is further supported by comparing the experimental and theoretical powder X-ray diffraction plots, which is also shown in Fig. 1a. The helium void fraction of TAMOF-1 was calculated to be 0.427.

**Table 1:** Porosity parameters of TAMOF-1

	Surface area (m^2^ g^−1^)	Pore volume (cm^3^ g^−1^)
Experiments^31^	980 ± 50	0.38 ± 0.02
Simulation	1080	0.37

To gain better understanding of the internal pore structure of the framework, the Pore Size Distribution (PSD) was also calculated and is plotted in Fig. 1b along with the accessible pore volume in Fig. 1c. The PSD shows three clear peaks, at 4.1, 6.3 and 7.8 Å. These peaks are calculated as void spheres of different sizes in the structure, which are represented by the color of the different regions of the pores in Fig. 1c. The void spheres along with the framework atoms of TAMOF-1 are visualized in Fig. S1.† It can be seen that the entire void space is an intricate interconnected network. The largest void space, corresponding to the two largest peaks of the PSD, consists of interwoven bended channels. Connected to these channels are smaller pockets of space that lie near the copper atoms of the framework. The copper atoms are aligned in triangles with sides of 6.7 Å and have an oxidation state of +2, which causes an electrostatic potential that will become important later when we begin to look at the adsorption of CO_2_. It is important to note that each of the smaller pockets are only connected from one side to the larger void space, making them less connected to the rest of the pore network (Fig. 1c). Creating this in-depth description of the pore network will prove vital in understanding the adsorption mechanisms occurring in TAMOF-1.

### 3.2 Pure component adsorption

**Fig. 2:**
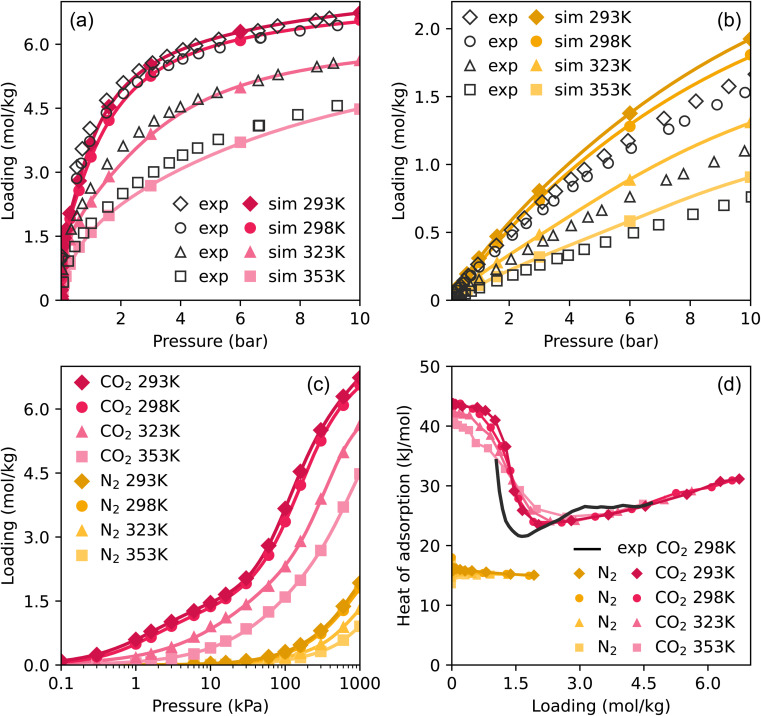
Experimental and simulated adsorption isotherms of (a) CO_2_ and (b) N_2_ at four temperatures. The colored points are the results of the MC-simulations and the drawn curves are the fitted isotherms. Simulated (c) isotherms and (d) heats of adsorption of CO_2_ and N_2_. The simulated heats of adsorption are computed using a fluctuation formula.^38^

To validate our method and force field for simulating adsorption isotherms, we compared experimental pure component isotherms to Monte Carlo simulations of CO_2_ and N_2_ from 0.1 to 10 bar at four different temperatures. These are shown in Fig. 2a and b, the same plots with a logarithmic pressure scale are shown in Fig. S2.† The results of the simulations show very good agreement with the experimental loadings, particularly in the case of CO_2_. In the case of N_2_, the simulations start to overestimate the loading somewhat at high pressures. Around the atmospheric pressure regime, which is the target for flue gas applications, the loading is well represented. These results show that our model is able to make excellent predictions on the adsorption of pure CO_2_ and N_2_ in TAMOF-1.

To compare the isotherms of CO_2_ and N_2_ directly, the isotherms are plotted together in Fig. 2c on a logarithmic scale, as differences in the shape of the isotherms become apparent. TAMOF-1 adsorbs CO_2_ much better than N_2_, because under ambient conditions, TAMOF-1 is predicted to have a CO_2_ loading of 3.4 mol kg^−1^ while N_2_ is scarcely adsorbed (0.28 mol kg^−1^). The absolute difference in adsorption of CO_2_ and N_2_ even increases when the pressure increases to 1000 kPa, with the predicted adsorbed amount being 6.5 and 1.8 mol kg^−1^ respectively.

We can use a review of Zhang *et al.* to compare the CO_2_ uptake of TAMOF-1 to other MOFs, which reports the uptakes of several high performing MOFs in Table 1.^61^ As a reference, TAMOF-1 has an uptake of 14.8 wt% and 87 cm^3^ cm^−3^ under the same conditions. The uptake capacity of TAMOF-1 is close to that of Cu-BTC, Mmen-CuBTTri and Bio-MOF-11, but it has considerably lower uptake than the other MOFs reported, particularly the high-performing M-MOF-74 series. TAMOF-1 has other advantages over these MOFs, particularly its high stability which makes it more suitable for practical applications.

Fig. 2c also shows a difference in the shape of the isotherms between CO_2_ and N_2_. The isotherms of CO_2_ show a plateau at intermediate pressures, most notably visible at 293 and 298 K. The isotherms of N_2_ do not show this shape, as the adsorption increases more linearly. The heats of adsorption of CO_2_ and N_2_, shown in Fig. 2d, reflect the differences between the isotherms. N_2_ shows a nearly constant heat of adsorption of around 16 kJ mol^−1^. The heat of adsorption at low loadings is more than twice as high for CO_2_, where at room temperature the initial heat of adsorption is 45 kJ mol^−1^, consistent with adsorption at lower pressures. As the loading of CO_2_ increases to 2.5 mol kg^−1^, the heat of adsorption decreases significantly to 24 kJ mol^−1^, after which it gradually begins to increase again.

This initial decrease in heat of adsorption for CO_2_ was demonstrated experimentally previously, and shows very close agreement with our simulated values (Fig. 2d). This stepwise adsorption mechanism in TAMOF-1 was suggested to be caused by the Cu^2+^-atoms providing initial binding sites for CO_2_.^34^ The lone pairs on the oxygen atoms of CO_2_ interact attractively with the positively charged copper atoms. The quadrupole moment and polarizability of CO_2_ are also much higher than those of N_2_, 2.8 and 1.5 times respectively, which explains the preferential adsorption of CO_2_ in TAMOF-1.^62^ The copper atoms in the TAMOF-1 framework form triangles that border small pockets of open space, corresponding to the pink regions in Fig. 1.

**Fig. 3:**
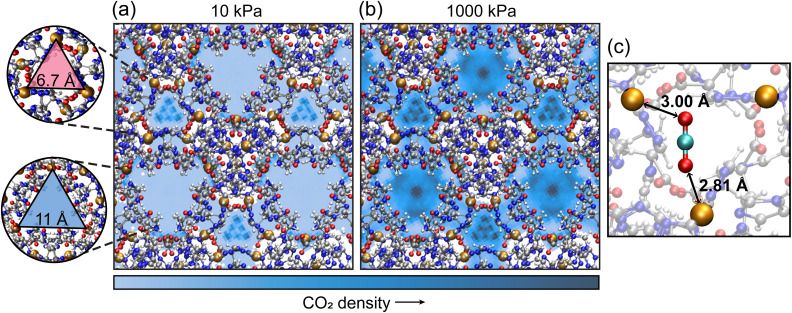
Distribution density plots of CO_2_ in the TAMOF-1 framework at 10 kPa (a) and 1000 kPa (b). The CO_2_-density is represented by the blue gradient, with the darker color corresponding to a higher CO_2_-density. On the left the two types of ‘channels’ are shown in a magnified view, with on top the channel with the triangular copper binding pockets and on the bottom the channel with larger void space. (c) The adsorption configuration of CO_2_ in a binding pocket, calculated by periodic DFT-calculations.

To elucidate the interaction between copper and CO_2_ in TAMOF-1, the adsorption configuration was calculated with periodic DFT-calculations and is shown in Fig. 3c. The oxygen atoms of CO_2_ lie aligned with two copper atoms, with distances of 2.81 and 3.00 Å. Along with the structural configuration, the adsorption energy was also calculated and gave an interaction energy of −53 kJ mol^−1^. A classical energy minimization at 0 K was also performed with RASPA using the same forcefield as employed in the Monte Carlo simulations. This gave an interaction energy of −49 kJ mol^−1^, which is in close agreement with the DFT-calculations.

To monitor the Cu–CO_2_ interactions along the isotherm, we can visualize CO_2_ in the binding pockets by rotating the framework and aligning the copper triangles in three directions, as shown in Fig. 3. Once rotated along one of these directions, channels with bigger triangular shaped pores appear parallel to the aligned copper triangles. Note that the pore network in Fig. 1c shows that the channels and windows are not actually triangularly shaped or linear and that the whole void structure is interconnected. Still, this description of two types of ‘channels’, visualized in the left part of Fig. 3, can be instrumental in distinguishing whether adsorbates are located inside or outside of the copper binding pockets.

To illustrate the role of the triangular copper binding pockets in the stepwise adsorption mechanism of CO_2_, the distribution plots of CO_2_ in TAMOF-1 at low and high pressure are shown in Fig. 3. In this view, the structure is aligned along one of the three directions in which the copper triangles lie. At 10 kPa, 86% of CO_2_ molecules are calculated to lie within 6.7 Å of an incenter of one of the copper triangles. Therefore, the three channels with the copper triangles show the highest density of CO_2_ in the framework, whereas the channels with larger pores possess far lower CO_2_ densities. When there is 6.4 mol per kg CO_2_ adsorbed at 1000 kPa, only 21% of the adsorbed CO_2_ molecules lie in the copper binding pockets. This indicates that the density of CO_2_ has shifted to the larger void spaces, since there were no more copper binding pockets left where CO_2_ was not already adsorbed. This is also visible in the distribution plot on the right of Fig. 3, since the previously almost empty space now possesses the highest CO_2_ distribution. These findings show that Monte Carlo simulations can provide detailed mechanistic insights into adsorption mechanisms.

### 3.3 Equilibrium mixture prediction

As the next step, the separation performance of TAMOF-1 of the main flue gas components is assessed. This is first done in equilibrium, making it possible to efficiently calculate several separation metrics over a wide pressure range. For this purpose, we used a numerical model based on IAST as implemented in the RUPTURA software, because it is several orders of magnitude faster than atomistic Monte Carlo simulations sampling from a mixture.^51^ The fitting parameters of the pure adsorption isotherms of CO_2_ and N_2_ at different temperatures are reported in Table S3.† The IAST calculations finished nearly instantaneously and show no significant deviations to the explicit Monte Carlo simulations, as Fig. S4† shows. IAST calculations are therefore suitable for further predicting adsorption from mixtures.

**Fig. 4:**
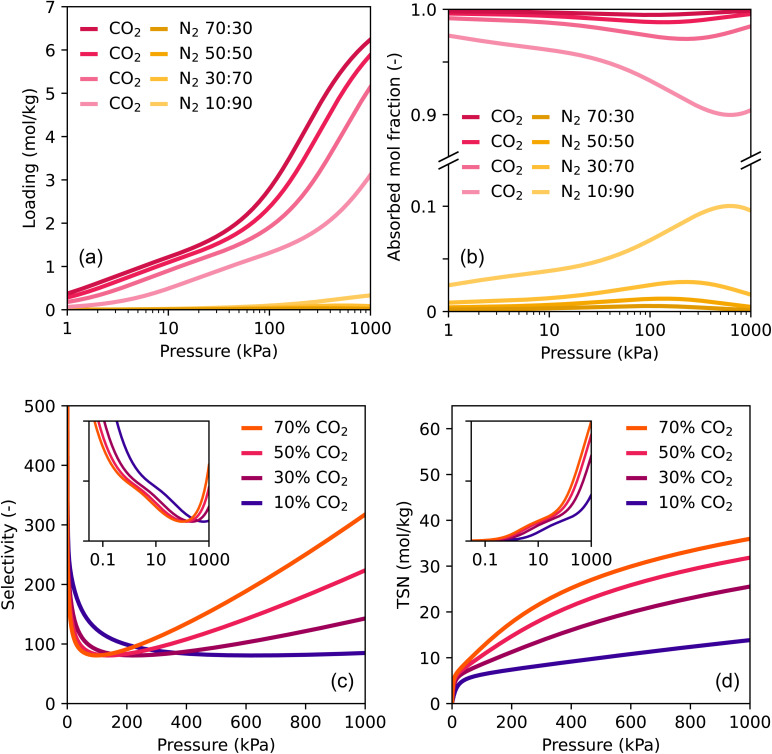
IAST predictions of the adsorption from CO_2_ : N_2_-mixtures of four bulk compositions at 298 K. (a) Loading, (b) adsorbed mole fraction, (c) selectivity and (d) trade-off between selectivity and uptake (TSN, see Methodology). The insets show the selectivity and TSN plotted in logarithmic scale.

Four CO_2_/N_2_-mixtures with different compositions were studied at 298 K, with the percentage of CO_2_ in the gas phase being 70, 50, 30 and 10 respectively. In Fig. 4a the predicted absolute loadings of the four mixtures are shown. CO_2_ is adsorbed in much higher quantities than N_2_ for all four mixtures, as was also the case for pure component adsorption. However, the loading of CO_2_ in TAMOF-1 decreases when the concentration of CO_2_ in the bulk mixture decreases. When we compare the loading from the pure isotherm to the 10% case for instance, the adsorbed loading decreases from 6.5 to 3.1 mol kg^−1^. This is expected, since a lower concentration CO_2_ corresponds to a lower partial pressure of CO_2_. In the case of 10% of CO_2_ in the bulk mixture, the partial pressure of CO_2_ is 1 bar, which corresponds to a pure component isotherm value of 3.5 mol kg^−1^, to which the predicted loading of 3.1 mol kg^−1^ from the mixture is only a slight decrease.

The adsorbed loading of N_2_ is almost reduced to zero when CO_2_ is mixed in. Even when 90% of N_2_ is still present in the bulk mixture, the loading at 10 bar is only 0.33 mol kg^−1^. This is a great reduction compared to the pure isotherm value of 1.62 mol kg^−1^. To compare the relative decrease in adsorption for both species, the adsorbed mole fractions of the four mixtures along the isotherms are shown in Fig. 4b. This property is also called the productivity, assuming the adsorbent is completely regenerated. When 30, 50 or 70% of N_2_ is present in the bulk gas, the adsorbed mole fraction is less than 0.05, resulting in negligible absolute amounts of N_2_ adsorbed in TAMOF-1. Although the adsorbed mole fraction of N_2_ increases when the bulk fraction increases to 0.9, it is still only at 10% at 1000 kPa.

The high adsorbed mole fractions of CO_2_ for all four compositions along the entire isotherm, translate into high selectivities, plotted in Fig. 4c; the inset shows the same curves but on a logarithmic pressure scale. At low pressures, the selectivities reach high numbers, reaching over 500. This is mainly due to the mole fraction of adsorbed N_2_ being close to zero at these pressures, which results in inflated selectivity numbers. The selectivities of all four mixtures then decrease to approximately 80, after which they start to increase again. This minimum lies at slightly different pressures between the four mixtures. The origin of this shift can be seen in Fig. 4b, where the peak of the adsorbed mole fraction of nitrogen shifts to higher pressures as the concentration of CO_2_ decreases. Strictly judging by the selectivity of the separation, at ambient conditions the 10%-mixture therefore performs the best. After reaching around 80, the selectivities start increasing again. The rate at which the selectivities increase increases as the concentration of CO_2_ rises. This is the result of N_2_ adsorbed fractions becoming close to zero again, so small differences in this number result in large differences in selectivity, without large differences in CO_2_-adsorption. For instance, between the 70 and 50%-mixture at 1000 kPa the difference in selectivity is about 100. However, the difference in the absolute loading of CO_2_ at this pressure is only 0.36 mol kg^−1^ (Fig. 4a). When it comes to comparing selectivities to previous reported values, data is scarce. The review of Zhang *et al.* does have calculated selectivities of the CO_2_/N_2_ separation of over 70 MOFs.^61^ However, due to the scarcity of data, the reported selectivities were calculated from pure component isotherms of CO_2_ and N_2_. This is a different method than the IAST-calculations we performed, and can lead to much lower selectivities. The lower selectivities calculated from pure isotherms result from an overestimation of N_2_ adsorbed in the MOF if the adsorbent possesses preferential adsorption sites for CO_2_. This is clearly the case for TAMOF-1, as well as other MOFs with open metal sites. Still, in order to have something to compare to, we can calculate the CO_2_/N_2_ selectivity of TAMOF-1 the same way. Using the pure component isotherm values, the selectivity value ends up to be around 40, which falls somewhere in the middle. However, as mentioned above, a selectivity value of 120 of TAMOF-1, calculated by IAST, is expected to be closer to reality. The same argument could be made for more MOFs however, so comparison of values remains difficult. Judging the separation performance on the basis of the selectivity as the sole metric is therefore not sufficient, especially if one adsorbate is hardly adsorbed from the mixture.

For this reason, we also calculated the trade-off between Selectivity and uptake (TSN, see Methodology), which is a product of the absolute uptake of CO_2_ and the logarithmic selectivity (eqn (3)). These are reported in Fig. 4d in linear scale, and in log scale in the inset. As the selectivities of all four mixtures and throughout the pressure range are sufficiently high, the main differences in TSN arise from differences in absolute loading of CO_2_. The logarithmic inset therefore shows the TSN curves having the same shape and trends as the loading curves. As this metric has not yet been used extensively, it is difficult to compare this performance of TAMOF-1 to other MOFs. One study calculated the TSN of the separation of 50 : 50 CO_2_ : CH_4_ of many MOFs at 10 bar and 298 K.^58^ At those conditions, the TSN was around 5 for most MOFs, while high-performing MOFs had TSNs between 10 and 20. TAMOF-1 appears to perform very well in comparison, as the 50 : 50 CO_2_ : N_2_ mixture shows a TSN of 30 at 10 bar. However, N_2_ uptake is lower than methane in TAMOF-1, so we can expect lower TSN values for natural gas separations. Nevertheless, it indicates that TAMOF-1 still exhibits an adequate high CO_2_-uptake, although the selectivity is very high for the CO_2_/N_2_ separation.

The influence of the temperature on the separation performance was also tested for the 10 : 90 CO_2_/N_2_, shown in Fig. S5.† When the temperature increases from 298 to 353 K, the absolute loading of TAMOF-1 of CO_2_ decreases by 2 mol kg^−1^, while the loading of N_2_ barely decreases. The adsorbed mole fraction of CO_2_ also decreases to a minimum of 0.8 at 3 bar, which leads to selectivities below 50. The TSN-numbers also decrease more than two-fold, which is expected as the selectivity and mainly the absolute loading show a significant decrease. This indicates the CO_2_/N_2_ separation performance of TAMOF-1 will be negatively affected by an raising the temperature, mainly caused by lower adsorption capacities of CO_2_ at higher temperatures.

As a general remark, Fig. 4 shows large differences for all properties along the pressure range. To retrieve all of these values experimentally along the whole pressure range is often not possible, because of time and cost constraints. The numerical modeling used in this work can very efficiently provide a lot of information on the separation performance. This provides a more complete picture on how a material might perform under different conditions.

### 3.4 Breakthrough curve modeling

**Fig. 5:**
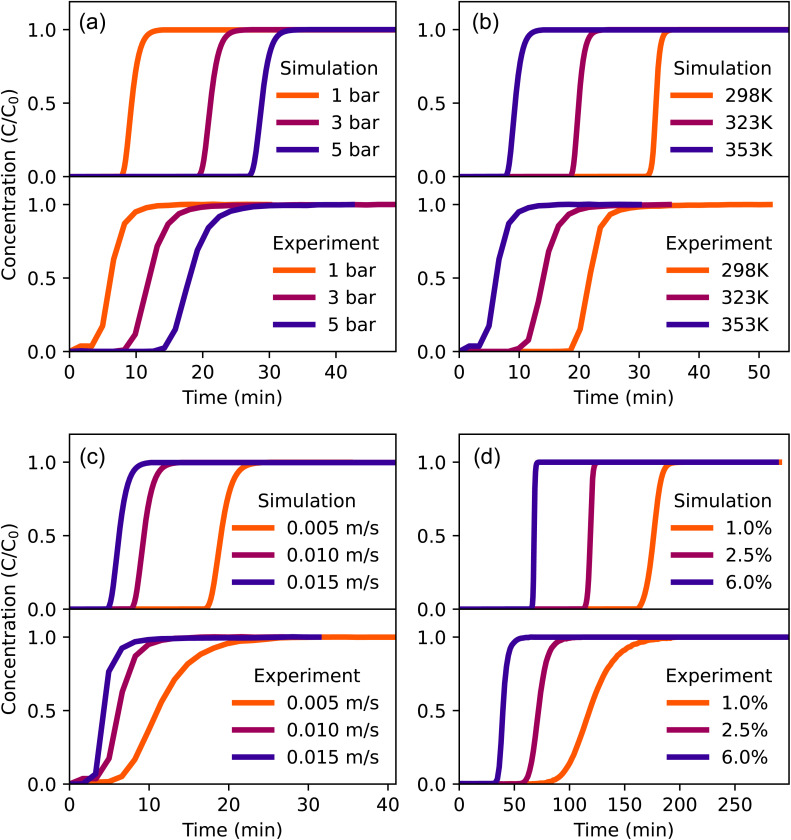
Simulated (top) and experimental (bottom) breakthrough curves of CO_2_ in helium. (a) Pressures at 353 K, 0.01 m s^−1^, 6% (b) temperatures at 1 bar, 0.01 m s^−1^, 6% (c) velocities at 353 K, 1 bar, 6% (d) concentrations at 298 K, 1 bar, 0.005 m s^−1^. *C*_0_ is the CO_2_-concentration at the inlet.

Predicting dynamic separation properties is the final important step of the TAMOF-1 performance evaluation, because it is important to incorporate kinetic effects and assess the scalability of an adsorption device. We therefore combined experiments and simulations to measure and predict breakthrough curves of the main flue gas components in TAMOF-1. We performed numerical breakthrough simulations with an isothermal model based that is very efficient in the prediction of breakthrough curves, as the longest simulation took around 1 hour. Combining experiments and simulations, we first compare breakthrough curves of CO_2_ in helium in a column with a length of 5.8 cm. The additional column conditions are listed in Table S5.† We varied the pressure, temperature, flow rate and concentration to test the response to different conditions, this is shown for both experiments and simulations in Fig. 5. Details on the studied conditions are listed in Table S6.†

The simulations were able to correctly predict the trends in the breakthrough times. To achieve this, an important note on the increasing breakthrough times with increasing pressure, shown in Fig. 5, must be made. As the pressure increases, the volumetric flow rate in the column will decrease because the molar rate stays constant. Therefore, the initial velocity input variable of the simulation needs to be decreased along with increasing pressure. In this way, the model can correctly match the experimentally increasing breakthrough times with increasing pressure. The calculation of the correct initial velocity in the column is done using the ideal gas law, explained in more detail in Section S4.3.† The trend of temperature is also well predicted by the simulations (Fig. 5 b): lower temperatures produce longer retention times for CO_2_, due to the higher adsorption capacity of TAMOF-1, which is confirmed by the isotherms. The time in which the inlet concentration of CO_2_ is reached at the outlet is predicted accurately for 323 and 353 K, with time differences of seconds. The agreement is worse for 298 K, with a difference of around 8 minutes. Changing the flow rate and CO_2_ concentrations result in similar behavior in the performance of the model (Fig. 5c and d). When moving to lower flow rates and concentrations, the time where equilibrium is reached (*C*/*C*_0_ = 1) is still predicted very accurately, but the simulated curves are much steeper than the experimental ones. The width of the mass transfer zone has many influencing factors, among which the mass transfer rate, axial dispersion, and heat transfer effects.^51^ Some studies indicate that heat transfer effects in particular are expected to play an important role in breakthrough experiments with CO_2_, due to the relatively high heat of adsorption of CO_2_.^63–65^ Incorporating heat transfer effects into the model would therefore likely improve these results.

**Fig. 6:**
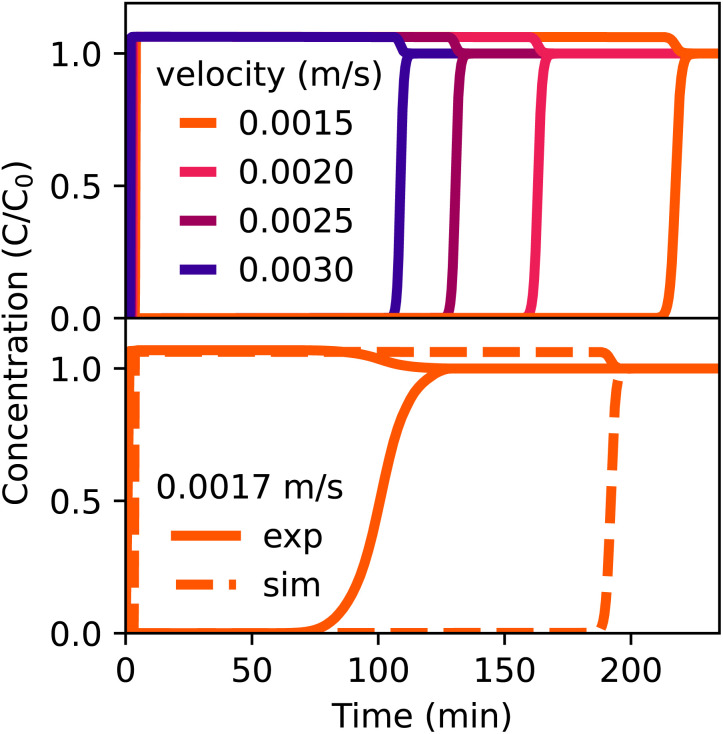
Bottom: simulated and experimental breakthrough curves of 6% CO_2_ and 94% N_2_ at 298 K, 1 bar and 0.0017 m s^−1^. Top: simulated curves with same conditions at different initial velocities. The retained curves is in all cases CO_2_, N_2_ leaves the column almost immediately.

A breakthrough experiment with CO_2_ in N_2_ was also performed, where the column and conditions were the same as in the helium system, except for the gas velocity, which was significantly lower (0.0017 m s^−1^). The conditions and parameters can be found in Table S7.† The bottom of Fig. 6 shows the experimental and simulated breakthrough curves of CO_2_ and N_2_ under these conditions. N_2_ exits the column almost immediately for both the experiment and simulation, because the adsorption capacity of TAMOF-1 for N_2_ is extremely low. Interestingly, CO_2_ is predicted to elute around 60 minutes later than is experimentally observed under these conditions, so the model performs much worse than the helium case. The large time differences are possibly caused by fluctuations in the gas velocity, to which the model is very sensitive at low velocities. To illustrate this, simulation results of different inlet velocities around the value of 0.0017 m s^−1^, although all still significantly lower than the previously used lowest velocity of 0.005 m s^−1^, are shown at the top of Fig. 6. Using an inlet velocity of 0.0025 m s^−1^ in the simulation gives the same saturation time (133 min) as the experiment with the lower flow rate. When the velocity is slightly reduced to 0.002 m s^−1^, this point shifts to 166 min. This shift now becomes even larger if it is further reduced to 0.0015 m s^−1^, where the point of saturation lies at 222 min. Therefore, these large absolute time differences between these velocities will make it more difficult to accurately predict the breakthrough times when the flow rate is this low. To investigate diffusion effects, the effective diffusion constants of CO_2_ in TAMOF-1 at different adsorbed loadings were calculated from MD-simulations, as described in Section S5.† The effective diffusion coefficients are in the range of 0.15 to 1.67 × 10^−6^ m^2^ s^−1^, as shown in Fig. S9.† During the MD-simulations, the CO_2_ molecules were moving between different adsorption sites, indicating that intraparticle diffusion is not expected to have an inhibiting influence on the separation. Additionally to the sensitivity to the low flow rate, there are of course more limitations to the simulation model as mentioned previously, resulting from the inherent approximations implemented, such as the isothermal condition and ideal gas approximation. However, because experiments also show N_2_ eluting immediately and simulations of CO_2_ in He at higher flow rates showed much better agreement, the numerical model at should also be suitable for simulating breakthrough curves of flue gas separations at higher flow rates.

**Fig. 7:**
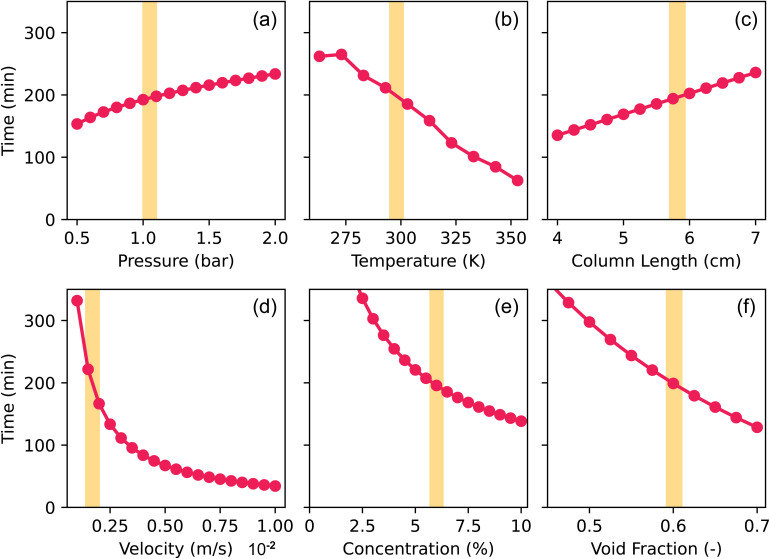
Time where equilibrium (*C*/*C*_0_ = 1) of CO_2_ is reached in the modeling of the breakthrough curves of CO_2_ and N_2_ in TAMOF-1. The (a) pressure, (b) temperature, (c) column length, (d) gas velocity, (e) CO_2_-concentration and (f) void fraction are varied, while keeping the other conditions fixed at the conditions of the experiment shown in Fig. 6 (shown with the yellow bands and listed in Table S7†).

To test this, the sensitivity of the model to certain conditions was mapped, as it influences the reliability and robustness of the predictions. Therefore, we used the system mentioned above of CO_2_ and N_2_ and varied one variable at a time, keeping the other variables fixed at the values of the experiment mentioned above (Table S7†). The times at which *C*/*C*_0_ of CO_2_ reached unity are plotted for each varied variable in Fig. 7, with the experimental conditions shown as yellow bands. The vertical axis is the same for all graphs, to easily compare the sensitivity of the model with respect to the variables. To test the effect of temperature, some additional isotherms were generated using a thermodynamical model.^57^ To validate this method, we compared the generated isotherms with the explicitly simulated isotherms in the range of 263–353 K in Fig. S3.† As the agreement between both methods was satisfactory, we proceeded to fit the generated isotherms of temperatures we had not simulated with Monte Carlo simulations to the multi-site Sips equations, of which the fitting parameters are in Table S4.†

The times where equilibrium (*C*/*C*_0_ = 1) of CO_2_ is reached show more or less linear relations with respect to pressure, temperature, column length, and void fraction (Fig. 7a–c and f). For the chosen range of pressure and column length the timings lie between 150 and 250 min, so the model is able to withstand small deviations of these variables. The timings of the temperature show more variation, but the range of 263–353 K is quite broad, so experimental fluctuations are not expected to cause a major decrease in the accuracy of the prediction. The same argument also applies to the column void fraction. As all of these variables show linear relations within these ranges, a different choice of standard condition of the variables (shown with the yellow bands) will not affect the sensitivity to fluctuations. However, flow rate and concentration affect the timings very differently (Fig. 7d and e). The initial velocity shows an exponential curve that increases rapidly when the velocities decrease below 0.0025 m s^−1^. At the low velocities, the model is therefore very sensitive to deviations of the flow rate. As the yellow band at 0.0017 m s^−1^ shows, the experimental flow rate lies in the steep part of the curve, making it much harder to make accurate predictions of the breakthrough times. The CO_2_–helium system used for the validation in Fig. 5 used higher gas velocities, from 0.005 m s^−1^ upward. These flow rates lie in a much flatter part of the curve, so the reliability of the predictions is less affected by possible experimental fluctuations or deviations. The curve of different concentrations also shows an exponential shape (Fig. 7e). At 2.5% CO_2_, the time has already increased to 335 min and decreasing the concentration to 1% even increases the time to 500 min. However, at 6% of CO_2_, the curve has already flattened significantly, so the model is much less sensitive in that regime. These tests are important to perform as they indicate where the model might have limitations to reliably predict breakthrough times as experimental conditions can be hard to determine precisely and remain constant.

**Fig. 8:**
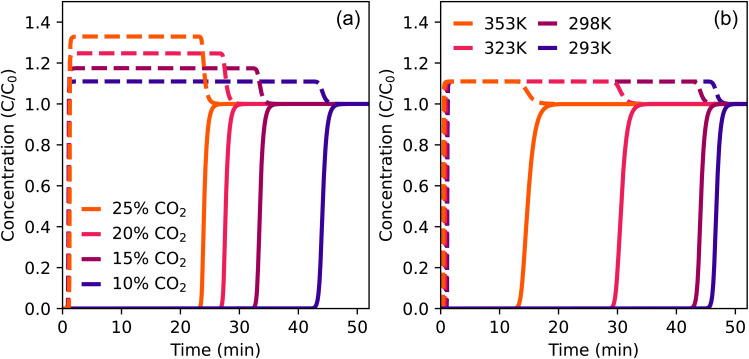
Simulated breakthrough curves of CO_2_ (solid) and N_2_ (dashed) at 1 bar and 0.005 m s^−1^. (a) Different concentrations of CO_2_ at 298 K (b) different temperatures of 10% CO_2_.

With the validation of CO_2_ in helium and the sensitivity tests of the model, we can now predict the breakthrough curves of flue gas separation with TAMOF-1. For this, the CO_2_ concentrations were modeled in the range of different flue gases at 298 K and the mixture of 10 : 90 CO_2_ : N_2_ at different temperatures, as shown in Fig. 8. Apart from the temperature and CO_2_-concentration, the same parameters were used as before (Table S7†), except for the initial velocity, which was increased to 0.005 m s^−1^.

All simulations show N_2_ leaving the column almost immediately, because it is hardly adsorbed by TAMOF-1. As the concentration of CO_2_ increases in the bulk gas, the adsorption capacity of TAMOF-1 is reached earlier, which results in a faster elution of CO_2_ (Fig. 8a). However, even at a CO_2_ concentration of 25%, which is higher than for most flue gases, CO_2_ can be efficiently separated from N_2_ with a breakthrough time of 23 min. Separation of CO_2_ and N_2_ is also possible in a wide temperature range (Fig. 8b). Although the adsorption capacity of TAMOF-1 decreases when the temperature increases from 298 to 353 K, CO_2_ still elutes separately from N_2_ after 13 minutes at 353 K, so TAMOF-1 is still able to separate CO_2_ from N_2_ at that temperature. These results confirm that TAMOF-1 is able to efficiently separate CO_2_ from N_2_ and therefore proves to be suitable for flue gas applications.

## 4 Conclusions

In this work, we combined experiments and simulations at the atomistic and numerical level to evaluate the adsorption and separation performance of TAMOF-1. In the interest of flue gas applications, we illustrated this by investigating CO_2_/N_2_ adsorption and separation. Simulated isotherms showed excellent agreement with experiments. The pure component isotherms showed a stepwise adsorption for CO_2_, accompanied by higher loadings and heats of adsorption than for N_2_. The preferential adsorption of CO_2_ was the result of binding sites near triangles of Cu^2+^ atoms, which lie dispersed through an intricate interconnected pore network of TAMOF-1. The CO_2_/N_2_ separation performance of TAMOF-1 of different compositions and temperatures over a wide pressure range could then be efficiently screened using numerical simulations. The modeled predictions showed TAMOF-1 preferentially adsorbs CO_2_, resulting in high adsorbed mole fractions, selectivities and trade-off between selectivity and uptake (TSN) numbers. Using the three metrics together along the entire pressure range can provide a wide overview of the separation performance. Breakthrough experiments and simulations confirmed this separation performance, as CO_2_ was retained in the column while N_2_ eluted almost immediately. To provide a comprehensive view of the simulation performance, the sensitivity of modeled breakthrough times to variations in column conditions was studied. At very low flow rates and CO_2_ concentrations, the model responds to small fluctuations with large increases in the breakthrough times of CO_2_. However, at most conditions, the simulations provided robust predictions and the simulated breakthroughs could accurately reproduce experimental trends as well as the times where *C*/*C*_0_ = 1. These findings confirm that TAMOF-1 is an excellent candidate for flue gas separation applications, because the Cu^2+^-atoms provide a favorable adsorption mechanism for CO_2_, resulting in good adsorption and excellent selectivities over N_2_ at ambient conditions. Additionally, combining experiments with both atomistic and numerical simulations is a great method to assess adsorption and separation performance, by exposing underlying adsorption mechanisms and efficiently screening many different operation conditions.

## Data availability

The data supporting this article have been included as part of the ESI.†

## Conflicts of interest

There are no conflicts to declare.

## Supplementary Material

TA-013-D5TA01362C-s001

## References

[cit1] Yaghi O. M., O'Keeffe M., Ockwig N. W., Chae H. K., Eddaoudi M., Kim J. (2003). Nature.

[cit2] Zhao X., Wang Y., Li D., Bu X., Feng P. (2018). Adv. Mater..

[cit3] Li J.-R., Kuppler R. J., Zhou H.-C. (2009). Chem. Soc. Rev..

[cit4] Furukawa H., Kim J., Ockwig N. W., O'Keeffe M., Yaghi O. M. (2008). J. Am. Chem. Soc..

[cit5] Furukawa H., Ko N., Go Y. B., Aratani N., Choi S. B., Choi E., Yazaydin A. O., Snurr R. Q., O'Keeffe M., Kim J., Yaghi O. M. (2010). Science.

[cit6] He Y., Krishna R., Chen B. (2012). Energy Environ. Sci..

[cit7] Wang B., Lv X.-L., Feng D., Xie L.-H., Zhang J., Li M., Xie Y., Li J.-R., Zhou H.-C. (2016). J. Am. Chem. Soc..

[cit8] Seo J. S., Whang D., Lee H., Jun S. I., Oh J., Jeon Y. J., Kim K. (2000). Nature.

[cit9] Li W., Zhang Y., Su P., Xu Z., Zhang G., Shen C., Meng Q. (2016). J. Mater. Chem. A.

[cit10] Kong L., Zou R., Bi W., Zhong R., Mu W., Liu J., Han R. P. S., Zou R. (2014). J. Mater. Chem. A.

[cit11] Lin Y., Lin H., Wang H., Suo Y., Li B., Kong C., Chen L. (2014). J. Mater. Chem. A.

[cit12] Xu H., He Y., Zhang Z., Xiang S., Cai J., Cui Y., Yang Y., Qian G., Chen B. (2012). J. Mater. Chem. A.

[cit13] Adil K., Belmabkhout Y., Pillai R. S., Cadiau A., Bhatt P. M., Assen A. H., Maurin G., Eddaoudi M. (2017). Chem. Soc. Rev..

[cit14] Liu R., Shi X., Wang C., Gao Y., Xu S., Hao G., Chen S., Lu A. (2021). ChemSusChem.

[cit15] Benoit V., Pillai R. S., Orsi A., Normand P., Jobic H., Nouar F., Billemont P., Bloch E., Bourrelly S., Devic T., Wright P. A., Weireld G. d., Serre C., Maurin G., Llewellyn P. L. (2016). J. Mater. Chem. A.

[cit16] Wells B. A., Chaffee A. L. (2011). Adsorption.

[cit17] Colón Y. J., Snurr R. Q. (2014). Chem. Soc. Rev..

[cit18] Wilmer C. E., Farha O. K., Bae Y.-S., Hupp J. T., Snurr R. Q. (2012). Energy Environ. Sci..

[cit19] Altintas C., Avci G., Daglar H., Azar A. N. V., Erucar I., Velioglu S., Keskin S. (2019). J. Mater. Chem. A.

[cit20] Bae Y., Snurr R. Q. (2011). Angew. Chem., Int. Ed..

[cit21] Haszeldine R. S. (2009). Science.

[cit22] Ding M., Flaig R. W., Jiang H.-L., Yaghi O. M. (2019). Chem. Soc. Rev..

[cit23] Rochelle G. T. (2009). Science.

[cit24] Sumida K., Rogow D. L., Mason J. A., McDonald T. M., Bloch E. D., Herm Z. R., Bae T.-H., Long J. R. (2012). Chem. Rev..

[cit25] McDonald T. M., Lee W. R., Mason J. A., Wiers B. M., Hong C. S., Long J. R. (2012). J. Am. Chem. Soc..

[cit26] Xiang S., He Y., Zhang Z., Wu H., Zhou W., Krishna R., Chen B. (2012). Nat. Commun..

[cit27] Benoit V., Chanut N., Pillai R. S., Benzaqui M., Beurroies I., Devautour-Vinot S., Serre C., Steunou N., Maurin G., Llewellyn P. L. (2018). J. Mater. Chem. A.

[cit28] Demessence A., D'Alessandro D. M., Foo M. L., Long J. R. (2009). J. Am. Chem. Soc..

[cit29] He H., Sun F., Aguila B., Perman J. A., Ma S., Zhu G. (2016). J. Mater. Chem. A.

[cit30] Caskey S. R., Wong-Foy A. G., Matzger A. J. (2008). J. Am. Chem. Soc..

[cit31] Corella-Ochoa M. N., Tapia J. B., Rubin H. N., Lillo V., González-Cobos J., Núñez-Rico J. L., Balestra S. R., Almora-Barrios N., Lledós M., Güell-Bara A., Cabezas-Giménez J., Escudero-Adán E. C., Vidal-Ferran A., Calero S., Reynolds M., Martí-Gastaldo C., Galán-Mascarós J. R. (2019). J. Am. Chem. Soc..

[cit32] Núñez-Rico J. L., Cabezas-Giménez J., Lillo V., Balestra S. R. G., Galán-Mascarós J. R., Calero S., Vidal-Ferran A. (2023). ACS Appl. Mater. Interfaces.

[cit33] González-Galán C., de Fez-Febré M., Giancola S., González-Cobos J., Vidal-Ferran A., Galán-Mascarós J. R., Balestra S. R. G., Calero S. (2022). ACS Appl. Mater. Interfaces.

[cit34] Capelo-Avilés S., de Fez-Febré M., Valestra S. R. G., Giancola S., Calero S., Galán-Mascarós J. (2025). Nat. Commun..

[cit35] Dubbeldam D., Calero S., Ellis D. E., Snurr R. Q. (2016). Mol. Simul..

[cit36] Macrae C. F., Sovago I., Cottrell S. J., Galek P. T. A., McCabe P., Pidcock E., Platings M., Shields G. P., Stevens J. S., Towler M., Wood P. A. (2020). J. Appl. Crystallogr..

[cit37] Sarkisov L., Bueno-Perez R., Sutharson M., Fairen-Jimenez D. (2020). Chem. Mater..

[cit38] Vlugt T. J. H., García-Pérez E., Dubbeldam D., Ban S., Calero S. (2008). J. Chem. Theory Comput..

[cit39] Mayo S. L., Olafson B. D., Goddard W. A. (1990). J. Phys. Chem..

[cit40] Rappe A. K., Casewit C. J., Colwell K. S., Goddard W. A., Skiff W. M. (1992). J. Am. Chem. Soc..

[cit41] García-Sánchez A., Ania C. O., Parra J. B., Dubbeldam D., Vlugt T. J. H., Krishna R., Calero S. (2009). J. Phys. Chem. C.

[cit42] Martín-Calvo A., García-Pérez E., García-Sánchez A., Bueno-Pérez R., Hamad S., Calero S. (2011). Phys. Chem. Chem. Phys..

[cit43] Lorentz H. A. (1881). Ann. Phys..

[cit44] Berthelot D. (1898). C. R. Acad. Sci..

[cit45] Darden T., York D., Pedersen L. (1993). J. Chem. Phys..

[cit46] Kühne T. D., Iannuzzi M., Del Ben M., Rybkin V. V., Seewald P., Stein F., Laino T., Khaliullin R. Z., Schütt O., Schiffmann F., Golze D., Wilhelm J., Chulkov S., Bani-Hashemian M. H., Weber V., Borštnik U., Taillefumier M., Jakobovits A. S., Lazzaro A., Pabst H., Müller T., Schade R., Guidon M., Andermatt S., Holmberg N., Schenter G. K., Hehn A., Bussy A., Belleflamme F., Tabacchi G., Glöβ A., Lass M., Bethune I., Mundy C. J., Plessl C., Watkins M., VandeVondele J., Krack M., Hutter J. (2020). J. Chem. Phys..

[cit47] Perdew J. P., Burke K., Ernzerhof M. (1996). Phys. Rev. Lett..

[cit48] Grimme S., Antony J., Ehrlich S., Krieg H. (2010). J. Chem. Phys..

[cit49] Grimme S., Ehrlich S., Goerigk L. (2011). J. Comput. Chem..

[cit50] VandeVondele J., Hutter J. (2007). J. Chem. Phys..

[cit51] Sharma S., Balestra S. R. G., Baur R., Agarwal U., Zuidema E., Rigutto M. S., Calero S., Vlugt T. J. H., Dubbeldam D. (2023). Mol. Simul..

[cit52] Sips R. (1948). J. Chem. Phys..

[cit53] Myers A. L., Prausnitz J. M. (1965). AIChE J..

[cit54] Polanyi M. (1963). Science.

[cit55] Polanyi M. (1932). Trans. Faraday Soc..

[cit56] Dubinin M. M. (1960). Chem. Rev..

[cit57] Stavarache F., Luna-Triguero A., Calero S., Vicent-Luna J. M. (2024). Chem. Eng. J..

[cit58] Cooley I., Boobier S., Hirst J. D., Besley E. (2024). Commun. Chem..

[cit59] Shah M. S., Tsapatsis M., Siepmann J. I. (2016). Angew. Chem., Int. Ed..

[cit60] González-Galán C., Madero-Castro R. M., Luna-Triguero A., Vicent-Luna J. M., Calero S. (2024). Sep. Purif. Technol..

[cit61] Zhang Z., Yao Z.-Z., Xiang S., Chen B. (2014). Energy Environ. Sci..

[cit62] D'Alessandro D., Smit B., Long J. (2010). Angew. Chem., Int. Ed..

[cit63] Serra-Crespo P., Berger R., Yang W., Gascon J., Kapteijn F. (2015). Chem. Eng. Sci..

[cit64] Fan Y., Kalyanaraman J., Labreche Y., Rezaei F., Lively R. P., Realff M. J., Koros W. J., Jones C. W., Kawajiri Y. (2015). Ind. Eng. Chem. Res..

[cit65] Mulgundmath V., Jones R., Tezel F., Thibault J. (2012). Sep. Purif. Technol..

