# Supplementary material for: TAMOF-1 for capture and separation of the main flue gas components
Source: J Mater Chem A Mater. 2025 Apr 29;13(22):16879–92. doi: 10.1039/d5ta01362c (PMC12059982; doi:10.1039/d5ta01362c)
Supplement: TA-013-D5TA01362C-s001 [file TA-013-D5TA01362C-s001.pdf]

Supplementary Information (SI) for Journal of Materials Chemistry A.
This journal is © The Royal Society of Chemistry 2025

Supporting Information: TAMOF-1 for Capture and Separation of the main Flue Gas Components

S. Gooijer, S. Capelo-Avilés, S. Sharma, S. Giancola, J.R. Galán-Mascaros, T.J.H. Vlugt, D. Dubbeldam, J.M. Vicent-Luna, S. Calero

Contents

S1 Characterization TAMOF-1 **S2**

S2 Pure Component Adsorption **S3**
S2.1 Experimental set-up S3
S2.2 Force Field Parameters S3
S2.3 DFT input parameters S4
S2.4 Isotherm fitting S4
S2.5 Adsorption in logarithmic scale S5
S2.6 Generating isotherms with RETMAP S6

S3 Mixture Adsorption Prediction **S8**
S3.1 Validation IAST S8
S3.2 IAST at different temperatures S9

S4 Breakthrough Curve Modeling **S10**
S4.1 Experimental set-up S10
S4.2 Run settings and column parameters S11
S4.3 CO_2 in helium S11
S4.4 CO_2 in N_2 . . . . . S12

S5 Diffusion **S13**

S1 Characterization TAMOF-1

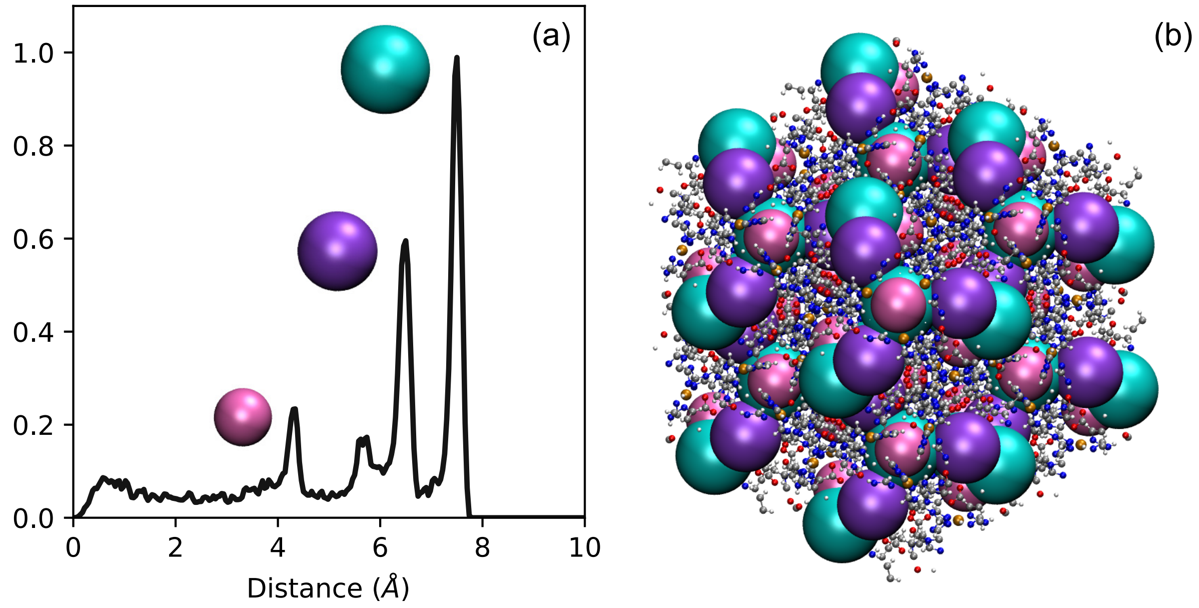

Figure S1: (a) PSD with the peaks represented by pink, cyan and purple spheres. (b) The pore-structure showing these spheres along with the framework of TAMOF-1.

S2 Pure Component Adsorption

S2.1 Experimental set-up

Adsorption isotherms for CO_2 and N_2 were measured using an Autosorb iQ (Quantachrome) at 298K and pressures up to 10 bar. Additionally, CO_2 and N_2 adsorption isotherms were collected at various temperatures ranging from 293–353K up to 10 bar. Before starting the analysis set, the sample is activated at 150°C for 15h under vacuum (10^{-06} torr). As the sample is hygroscopic, a degassing is performed at 120°C for 2h between each measurement step to eliminate traces of moisture by contact with air.

S2.2 Force Field Parameters

Monte Carlo simulations of 10000 initialization and 50000 production cycles were performed with the RASPA software.[1] During each cycle, N trial moves were performed, with N being the number of adsorbate molecules present. Every 1000 cycles the adsorption properties were printed and every 2000 cycles the coordinates of the adsorbate molecules were saved. For CO_2 and N_2, a fugacity coefficient of 1 was used. The translation, rotation and reinsertion probability was set to 0.5 and the swap probability to 1.0. 2 x 2 x 2 unit cells of TAMOF-1 were used to avoid molecules interacting with the same particles multiple times. The Ewald summation method was used for the evaluation of the Coulombic potential.[2] Shifted Lennard-Jones potentials without tail corrections with a cutoff of 12Å were used. Lorentz-Berthelot mixing rules were used to compute cross-interaction terms.[3–5] The Lennard-Jones parameters of the framework of TAMOF-1 were taken from the UFF (metal) and DREIDING (non-metal) force field.[6,7] The partial charges were calculated with the charge equilibration method implemented in RASPA.[8] The Lennard-Jones parameters along with the partial charges of the framework atoms are given in Table S1. For CO_2 and N_2 the Lennard-Jones parameters, geometries and partial charges were taken from existing models.[9,10] Both are rigid models, with a C-O bond length of 1.149 Å for CO_2 and a N-N bond length of 1.1 Å for N_2. The Lennard-Jones parameters along with the partial charges for the adsorbates are listed in Table S2.

Table S1: Lennard-Jones parameters and partial charges of the TAMOF-1 framework.

**atom**	**ϵ/k_B (K)**	**σ (Å)**	**q (e^-)**	**atom**	**ϵ/k_B (K)**	**σ (Å)**	**q (e^-)**
C1	47.856	3.473	-0.001	H2	7.649	2.846	0.002
C2	47.856	3.473	0.164	H3	7.649	2.846	0.040
C3	47.856	3.474	0.140	H4A	7.649	2.846	0.138
C4	47.856	3.474	-0.289	H4B	7.649	2.846	0.157
C5	47.856	3.474	0.193	H5	7.649	2.846	0.235
C6	47.856	3.474	-0.062	H6	7.649	2.846	0.069
C7	47.856	3.474	0.197	H7	7.649	2.846	-0.033
C8	47.856	3.474	0.373	N1	38.949	3.263	-0.182
Cu1	2.5161	3.114	1.029	N2	38.949	3.263	-0.264
O1	48.158	3.033	-0.327	N3	38.949	3.263	-0.146
O2	48.158	3.033	-0.352	N4	38.949	3.263	-0.302
H1	7.649	2.846	0.076	N5	38.949	3.263	-0.333

Table S2: Lennard-Jones parameters and partial charges of the adsorbates. Com denotes the center of mass of N_2.

**atom**	ϵ/k_B **(K)**	σ **(Å)**	**q (e^-)**
O (CO_2)	85.671	3.017	-0.3256
C (CO_2)	29.933	2.745	0.6512
N (N_2)	38.298	3.306	-0.405
com (N_2)	-	-	0.810

S2.3 DFT input parameters

Periodic DFT-calculations were performed using the Quickstep module of the CP2K-package.[11] The Perdew-Burke-Ernzerhof (PBE) functional with Grimme's dispersion corrections (D3) was used.[12–14] The DZVP-MOLOPT basis sets were used for the non-metallic atoms, while for copper the short-ranged equivalent (DZVP-MOLOPT-SR) was used.[15] An auxiliary plane waves basis set with a cutoff energy of 750 Ry was mapped unto a 5-level grid with a relative cutoff energy of 60 Ry. GTH-pseudopotentials were used to describe the effect of the core electrons.[16–18] The calculations were performed spin-polarized, with the system having a multiplicity of 13. The orbital transformation method was employed, using the FULL-ALL preconditioner and an energy gap set to 0.001. The target accuracy for the SCF-convergence was set to 1.0E-6.

First, a cell optimization of the unit cell of TAMOF-1 containing 564 atoms was performed, where the positions of the atoms as well as the unit cell dimensions were optimized. Then, the cell dimensions were fixed and a geometry optimization of CO_2 inside the TAMOF-1 framework was performed. The BFGS optimizing algorithm was used for both the cell and the geometry optimization.

S2.4 Isotherm fitting

The isotherms were fitted using the RUPTURA software.[19] Simulated isotherms were used as input and either 2 or 3 sites were used in the Sips equation (Equation 1) to achieve the optimal fit of the data. The resulting parameters are shown in Table S3. The fits are plotted along with the raw data in Figure 2 of the main text.

$$q(p) = \sum_i q_i^{\text{sat}} \frac{(b_i p)^{1/\nu_i}}{1 + (b_i p)^{1/\nu_i}} \tag{1}$$

Here $q(p)$ is the absolute loading of the adsorbed phase, q_i^{sat} the saturation loading, p the pressure and b_i and ν_i the affinity and Henry coefficients respectively.

Table S3: Parameters of the fit of simulated isotherms of CO_2 and N_2 at different temperatures.

T (K)	CO_2			N_2		
	q_{sat}	b	ν	q_{sat}	b	ν
263	1.587	0.004	1.100	2.873	$2.191 \cdot 10^{-06}$	1.040
	2.839	$4.581 \cdot 10^{-06}$	1.051	1.443	$1.160 \cdot 10^{-06}$	0.675
	3.801	$2.962 \cdot 10^{-05}$	0.537			
293	3.667	$9.364 \cdot 10^{-06}$	0.603	2.597	$1.380 \cdot 10^{-06}$	0.984
	2.937	$1.176 \cdot 10^{-06}$	1.122	0.746	$1.102 \cdot 10^{-06}$	0.422
	1.585	0.001	1.045			
298	3.114	$6.609 \cdot 10^{-06}$	0.606	2.244	$1.620 \cdot 10^{-06}$	0.901
	3.761	$4.455 \cdot 10^{-06}$	1.662	0.737	$1.028 \cdot 10^{-06}$	0.456
	0.876	0.001	0.777			
323	2.888	$2.841 \cdot 10^{-06}$	0.520	1.588	$1.155 \cdot 10^{-06}$	0.512
	3.446	$1.910 \cdot 10^{-05}$	1.459	0.450	$5.976 \cdot 10^{-06}$	0.842
353	1.931	$1.186 \cdot 10^{-06}$	0.522	0.564	$1.131 \cdot 10^{-06}$	0.308
	4.548	$4.178 \cdot 10^{-06}$	1.357	0.797	$2.164 \cdot 10^{-06}$	0.846

S2.5 Adsorption in logarithmic scale

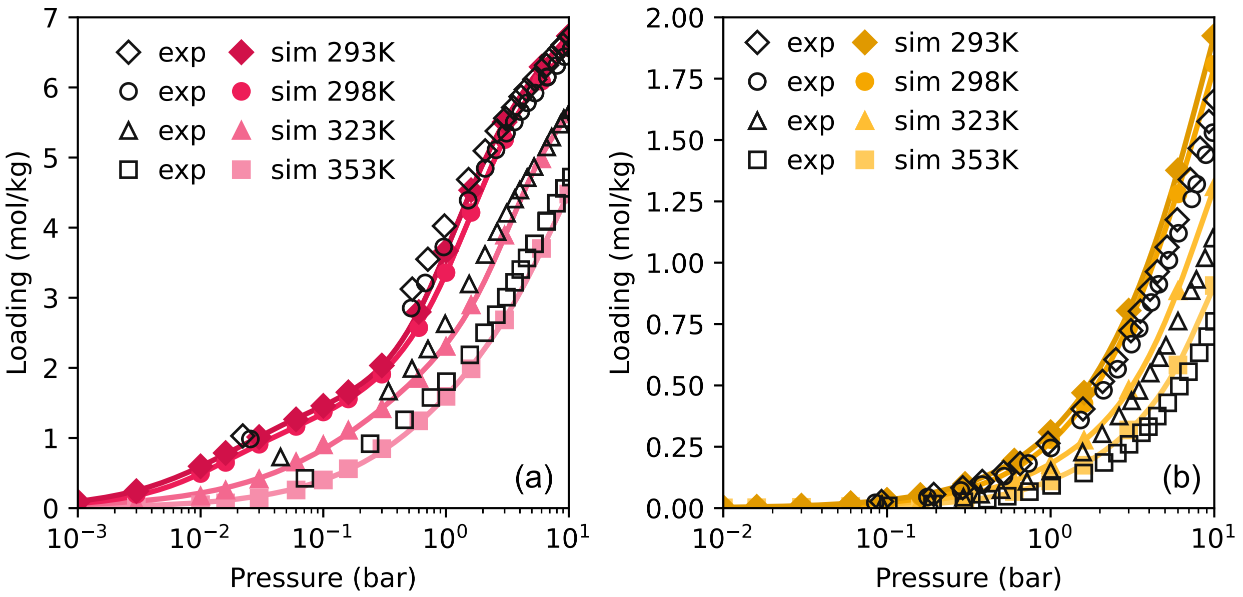

Figure S2: Experimental en simulated adsorption isotherms of (a) CO_2 and (b) N_2 at four temperatures with a logarithmic pressure scale. The colored points are the results of the MC-simulations and the drawn curves are the fitted isotherms.

S2.6 Generating isotherms with RETMAP

To quickly generate isotherms of many different temperatures, we used a mathematical model based on the adsorption potential theory[20–22]. According to this theory, for each adsorbate-adsorbent pair, there exists a characteristic curve. The characteristic curve is the relation between the adsorption potential and the pore filling. It does not depend on the temperature, which makes it possible to generate many different isotherms directly from the characteristic curve. The used mathematical model constructs the characteristic curve from one input isotherm and subsequently generates isotherms at other temperatures. This model has been published and is implemented in the RETMAP-software.[23] In order to construct the characteristic curve, the vapor saturation pressure and the adsorbate density within the cavities need to be modeled. The model uses the critical isochore as an approximation for the saturation pressure and Hauer's method for the adsorbate density.[24,25]

We used the isotherms of CO_2 and N_2 at 298K to generate isotherms in the range of 263-353K with the RETMAP software.[23] As validation, we compare the generated isotherms of CO_2 with our explicitly calculated isotherms in Figure S3. The shape of the isotherms are well reproduced by the RETMAP-method. The generated isotherms of the temperatures which were not explictly calculated by MC-simulations were fitted to the multi-site Sips Equation (Equation 1). The parameters of these fitted isotherms are shown in Table S4. These were used in the calculation of breakthrough curves at different temperatures in Figure 7b.

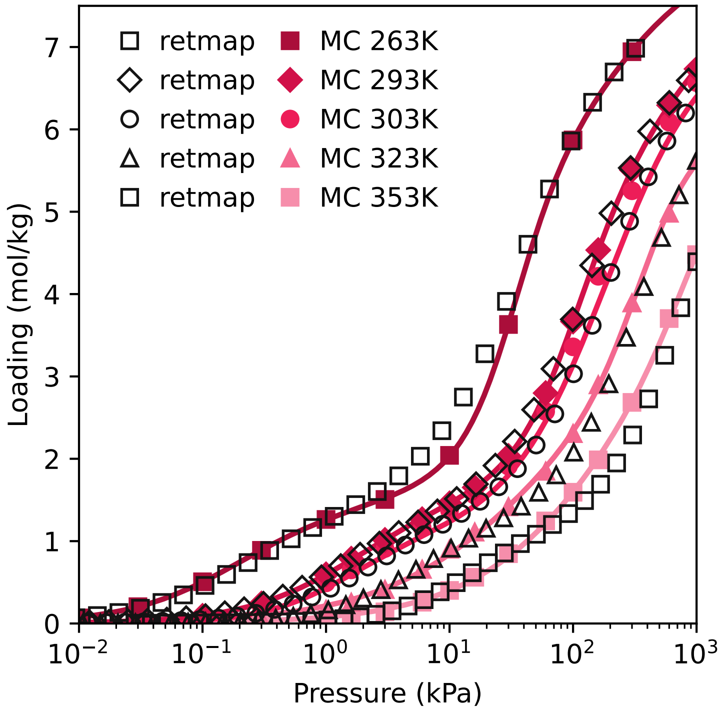

Figure S3: Comparison of the calculated adsorption isotherms of CO_2 in TAMOF-1 at five different temperatures with the generated isotherms using the RETMAP software[23].

Table S4: Parameters of the fit of isotherms calculated with the RETMAP software of CO_2 and N_2 at different temperatures.

T (K)	CO_2			N_2		
	q_{sat}	b	ν	q_{sat}	b	ν
273	5.870	$1.607 \cdot 10^{-05}$	0.848	2.092	$2.490 \cdot 10^{-06}$	0.589
	1.485	0.002	1.051	0.480	$1.829 \cdot 10^{-05}$	0.795
283	6.060	$1.079 \cdot 10^{-05}$	0.880	1.960	$1.716 \cdot 10^{-06}$	0.567
	1.295	0.001	0.911	0.713	$8.403 \cdot 10^{-06}$	0.829
313	6.167	$3.784 \cdot 10^{-06}$	0.889	0.752	$3.565 \cdot 10^{-06}$	0.795
	1.025	0.001	0.737	1.824	$9.664 \cdot 10^{-07}$	0.534
333	3.918	$1.899 \cdot 10^{-06}$	0.575	0.210	$7.607 \cdot 10^{-06}$	0.436
	2.540	$2.304 \cdot 10^{-05}$	1.397	2.154	$8.436 \cdot 10^{-08}$	0.556
343	2.990	$1.547 \cdot 10^{-06}$	0.463	$1.647 \cdot 10^{-16}$	0.001	1.077
	3.419	$7.597 \cdot 10^{-06}$	1.530	3.384	$4.660 \cdot 10^{-07}$	0.811

S3 Mixture Adsorption Prediction

S3.1 Validation IAST

To test the performance of numerical Ideal Adsorption Solution Theory (IAST) simulations to predict adsorption from mixtures, we performed an atomistic Monte Carlo simulation sampling from a mixture of CO_2 and N_2 with a mole fraction of 1:1. The same force field set-up was used as described in Section S2.2, with an additional trial move of an identity swap with a probability set to 0.5. The comparison of both simulations is shown in Figure S4, which shows that both yield the same mixture isotherms.

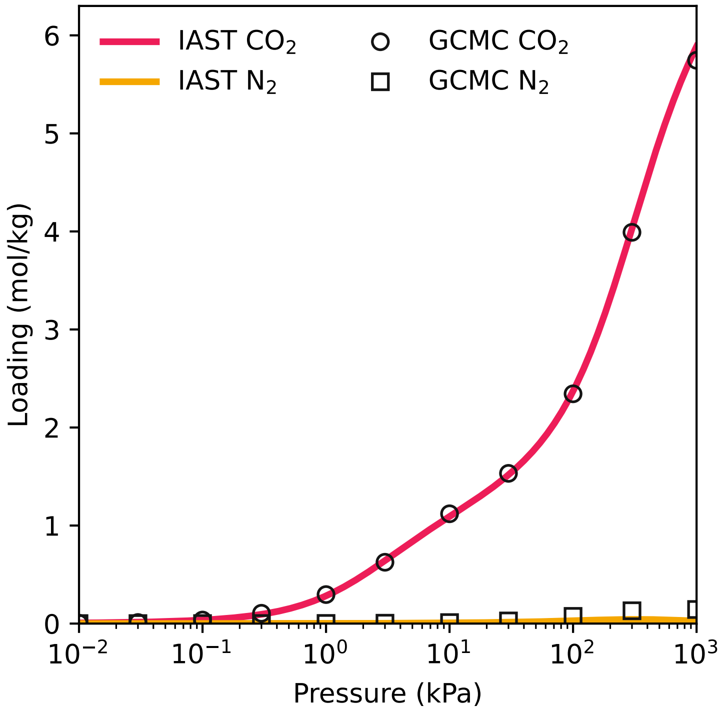

Figure S4: Mixture prediction of the adsorption of from a 1:1 CO_2:N_2 mixture at 298K. This is calculated with the IAST method (solid) and explicit MC-simulations (markers).

S3.2 IAST at different temperatures

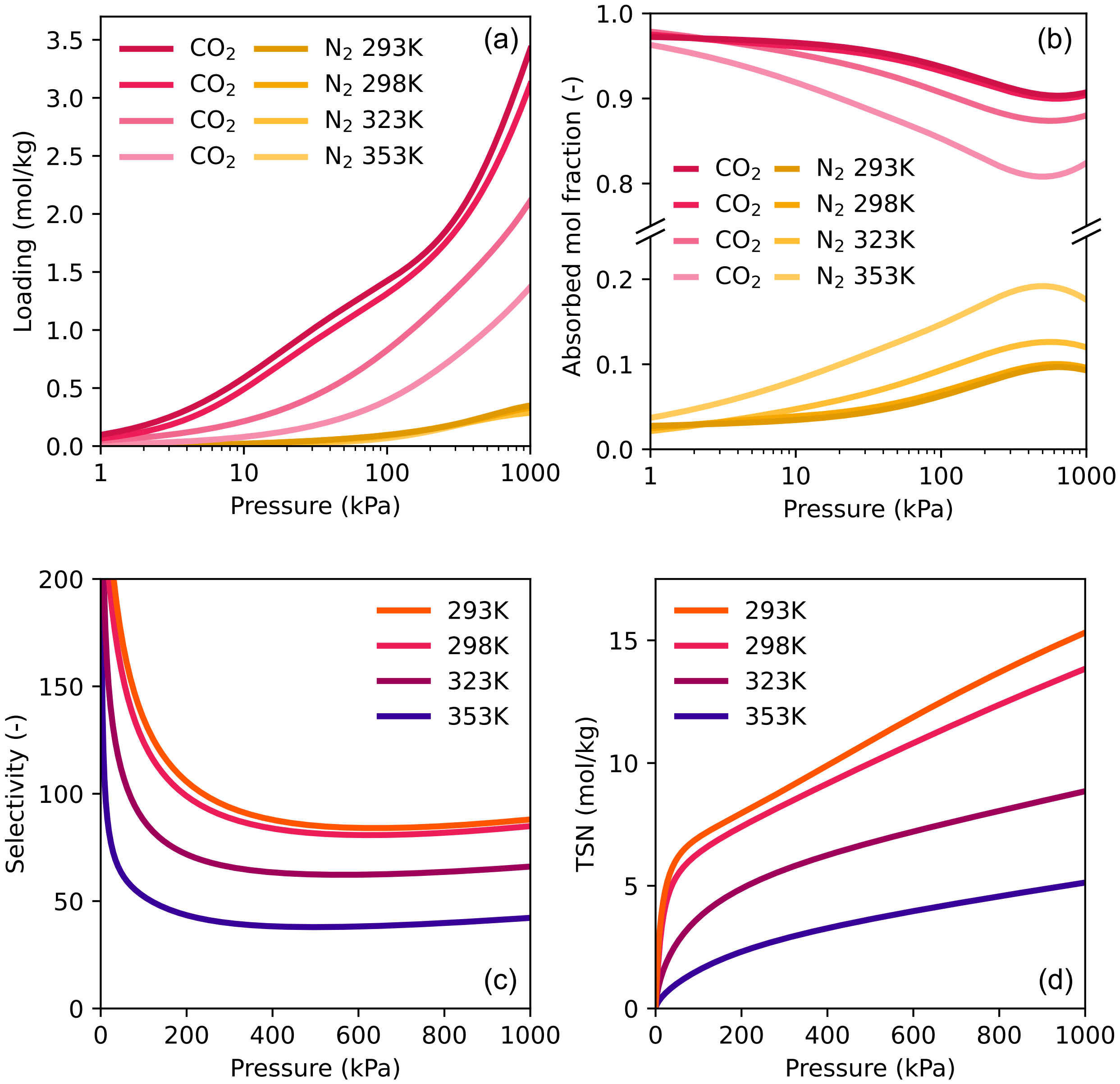

Figure S5: IAST predictions of the adsorption from 10:90 CO_2:N_2-mixtures at four temperatures. (a) Loading, (b) Adsorbed Molar Fraction, (c) Selectivity and (d) Tradeoff between Selectivity and uptake (TSN, see Methodology).

S4 Breakthrough Curve Modeling

S4.1 Experimental set-up

We used a fixed bed column with 1-inch (1.95 cm inner diameter) packed with 10.2 g (7.88 g dehydrated) of TAMOF-1 powder. The adsorbent bed height (5.3–5.8 cm) yielded a height-to-diameter ratio of 3–4. Glass wool filled the remaining column volume (Figure S6). The columns configuration parameters are detailed in the table S5. TAMOF-1 was activated in situ at 393K (1 K/min) under vacuum (10^{-2} mbar) for 15 hours.

Breakthrough measurements were performed using the experimental set-up shown in Figure S7. Gas cylinders of CO_2 (Air Liquide, 99.998%), N_2 (Praxair, 99.999%) and He (Linde, 99.999%) were used. Upstream of the separation module, calibrated mass flow controllers (Bronkhorst EL-FLOW) regulated the inlet gas flow rates and compositions. Downstream, a manometer and backpressure controller (Bronkhorst EL-PRESS) maintained the desired pressure. A second manometer, positioned upstream of the bed, monitored the inlet pressure when not under pressure control. All pressures are reported in absolute bar, unless otherwise stated. The separation module was heated by a silicone heating wire (Ø 3 mm FOR-FLEX NORMAL, Electricfor) rolled around the column. A K-type thermocouple (Thermocoax) inserted in the middle of the bed, and a temperature controller (Watlow EZ-Zone) maintained the desired temperature. The column outlet stream was analyzed online by a micro gas chromatograph (Agilent MicroGC 490) equipped with a Molsieve MS5A column (Ar carrier gas, 99.999% purity) and a Poraplot U column (He carrier gas, 99.999% purity), both using thermal conductivity detectors (TCD).

Table S5: Column configuration parameters.

Parameter	Value
Temperature (°C)	25
Column length (cm)	5.3–5.8
Column inner diameter (cm)	1.95
Bulk density (kg m^{-3})	680
Column void fraction	0.58

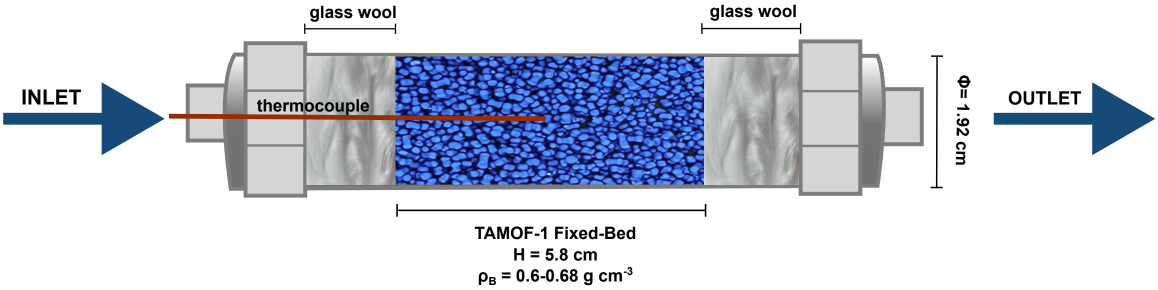

Figure S6: TAMOF-1 column configuration.

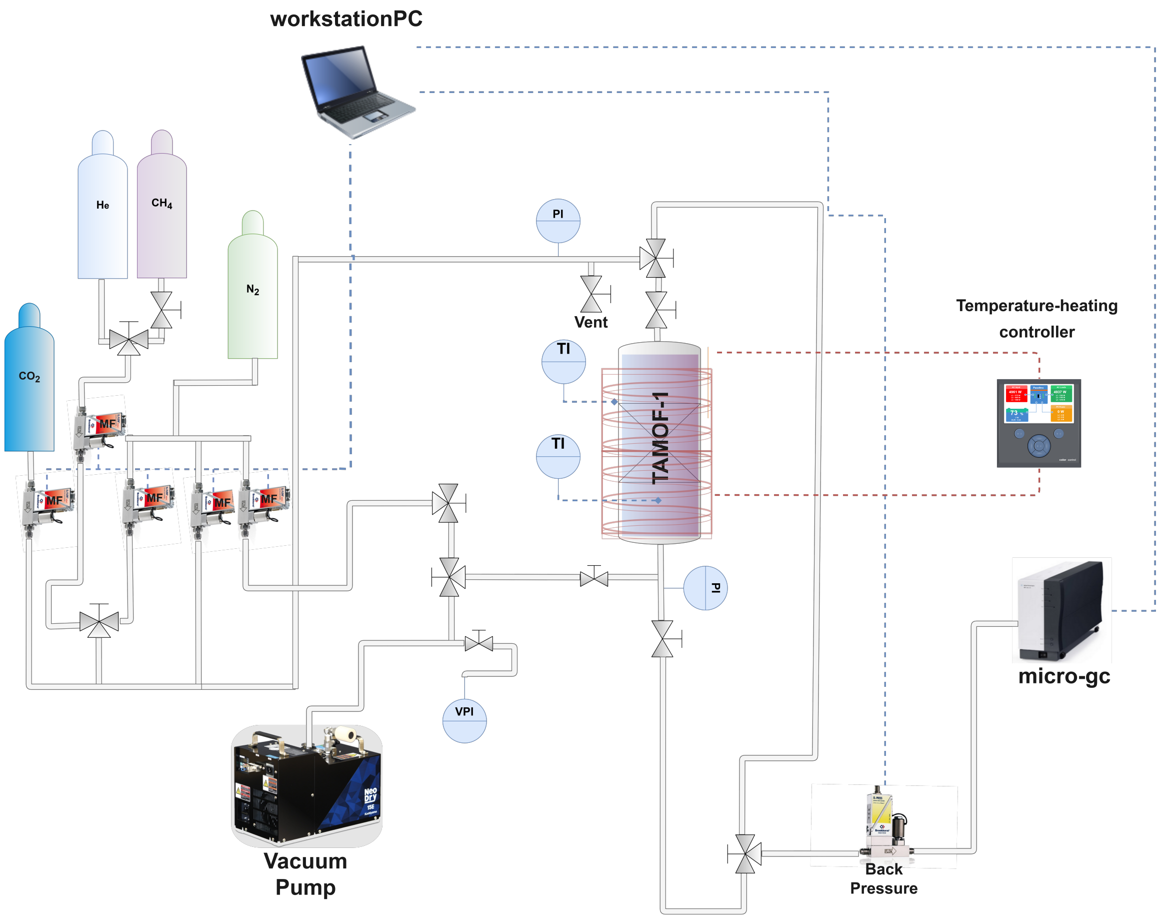

Figure S7: Experimental set-up of the breakthrough measurements.

S4.2 Run settings and column parameters

The breakthrough curves were modeled with the RUPTURA software.[19] The time step was set to 0.0005 s and every 10000 steps the data was printed. The number of grid points used was set to 100. As column parameters, the column length was 0.058 m, column void fraction was 0.58 and the particle density 1153.80 kg/m^3.

S4.3 CO_2 in helium

To validate the modeling of breakthrough curves, breakthrough experiments of CO_2 in helium were performed under different conditions. The temperature, pressure, flow rate and concentration were varied with the settings listed in Table S6. It is important to note the difference between the experimental feed gas velocity and the initial velocity used in the simulations at higher pressures. In the simulations, we need to input the initial velocity of the gas as it enters the column. In our experiments, the flow rate is set before the gas stream is pressurized. When the pressure of the gas stream increases, the actual velocity inside the column decreases as the concentration is fixed. The

actual initial velocity of the column can then be calculated with the ideal gas law (Equation 2).

$$P\dot{V} = \dot{n}RT \tag{2}$$

With P the pressure, $\dot{V}$ the flow rate, $\dot{n}$ the molar flow rate, R the gas constant and T the temperature. As the molar flow rate remains constant, the actual flow rate as it enters the column can be calculated with Equation 3. Knowing the diameter of the column, the flow rate can then be converted to the actual gas velocity as it enters the column, which is used as an input variable in the simulation.

$$P_1\dot{V}_1 = P_2\dot{V}_2 \tag{3}$$

Table S6: Conditions of the experimental and simulated breakthroughs of CO_2 in helium.

**Concentration** (%)	**Temperature** (K)	**Pressure** (bar)	**Exp. feed gas velocity** (m/s)	**Sim. initial gas velocity** (m/s)
6	298, 323, 353	1	0.005	0.005
6	298, 323, 353	3	0.005	0.0017
6	298, 323, 353	5	0.005	0.001
6	298, 323, 353	1	0.010	0.010
6	298, 323, 353	3	0.010	0.0033
6	298, 323, 353	5	0.010	0.002
6	298, 323, 353	1	0.015	0.015
6	298, 323, 353	3	0.015	0.005
6	298, 323, 353	5	0.015	0.003
1, 2.5, 6	298	1	0.005	0.005

S4.4 CO_2 in N_2

For the system with CO_2 in N_2, the used conditions are given in Table S7. These are also the values of the yellow bands displayed in Figure 7.

Table S7: Conditions of the experimental and simulated breakthroughs of CO_2 in N_2.

**Parameter**	**Value**
CO_2 concentration (%)	6
Temperature (K)	298
Pressure (bar)	1.05
Initial velocity (m/s)	0.0017
Column length (m)	0.058
Column void fraction (-)	0.58

S5 Diffusion

Molecular Dynamic (MD) simulations were performed using the RASPA software package to calculate self-diffusion constants of CO_2 in TAMOF-1 at different adsorbed loadings.[1] The same force field as for the Monte Carlo simulations was used, with the force field parameters given in Section S2.2. First, 10000 Monte Carlo initialization steps are performed, with random translation and rotation moves enabled. After 10000 equilibration steps with a timestep of 0.5 fs, a production run of 50 ns was performed in the NVT ensemble at 298K, from where the mean-square displacement (MSD) is calculated. The MSDs of five different loadings are plotted against time in log-log scale in Figure S8. The region of time where the slope of the curves plotted in log-log scales is close to 1 is shown with vertical lines. The slope of the MSDs in linear scale between these times were determined with linear regression.

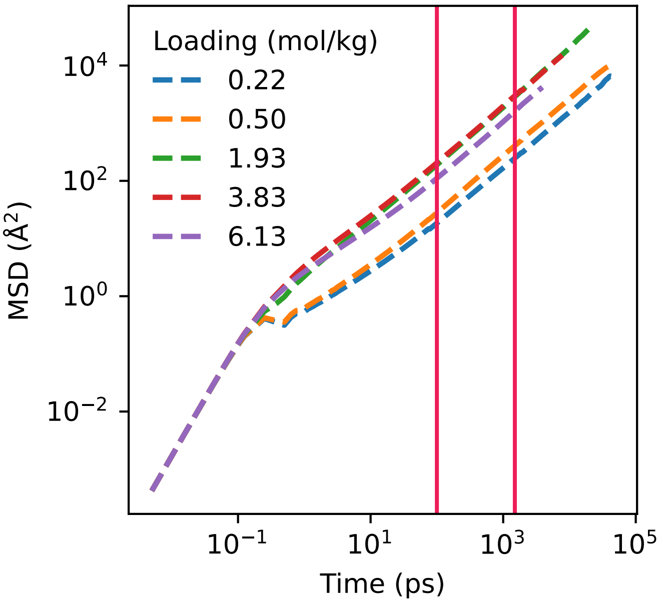

Figure S8: Mean Square Displacements (MSD) of CO_2 in TAMOF-1 at 298K of five different adsorbed loadings plotted against time in the log-log scale. The vertical lines indicate the region in which the slope of the curve in the log-log scale is close to 1. This is also the region of which the slope in linear scale was calculated to determine the self-diffusion coefficients.

The self-diffusion coefficient can be related to the MSD with Equation 4.

$$<x^2(t)> = 2dD_s t \tag{4}$$

Where d is the dimensionality of the pore, Ds the self-diffusion coefficient and t time. For convenience we assume that self-diffusion coefficients can be approximated to the Knudsen diffusion coefficients (D_k) under certain conditions, such in narrow pores where the interactions with the walls governs the diffusion of the molecules. With this assumption, the effective diffusion coefficients (D_{eff}) can then be calculated using $D_s \approx D_k$ and the molecular diffusion coefficients (Dm). The

molecular diffusion coefficients can be received from experimental data. The molecular diffusion coefficient for CO_2 at ambient conditions, taken from Walker et al., is 0.16 cm^2/s.[26] Effective diffusion coefficients can be calculated using Equation 5.

$$\frac{1}{D_{eff}} = \frac{1}{D_s} + \frac{1}{D_m} \tag{5}$$

The effective diffusion coefficients of different loadings of CO_2 are plotted in Figure S9, along with their respective heats of adsorption. The diffusion coefficients fall in the range of 0.15 to 1.67·10-6 m^2/s. Interestingly, the diffusion coefficients mirror the trend in heat of adsorption when the CO_2 loading increases.

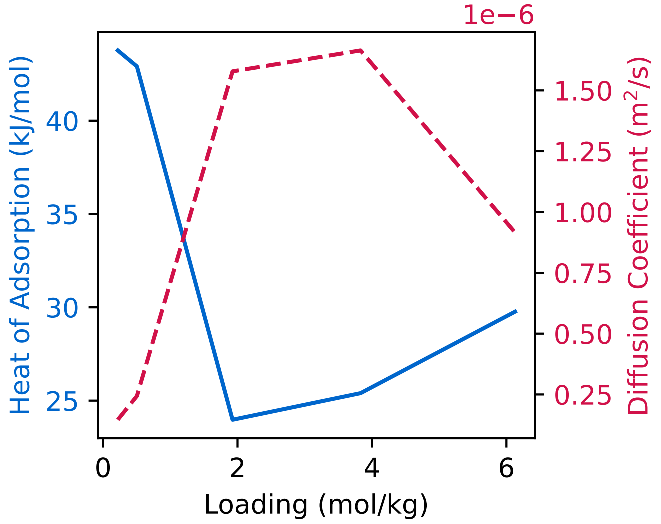

Figure S9: Effective diffusion coefficients of CO_2 in TAMOF-1 at 298K at different adsorbed loadings along with their respective heats of adsorption.

References

[1] D. Dubbeldam, S. Calero, D. E. Ellis and R. Q. Snurr, *Mol. Simul.*, 2016, **42**, 81–101.

[2] T. Darden, D. York and L. Pedersen, *J. Chem. Phys.*, 1993, **98**, 10089–10092.

[3] H. A. Lorentz, *Ann. Phys.*, 1881, **248**, 127–136.

[4] D. Berthelot, *Comptes. Rendus. Acad. Sci.*, 1898, **126**, 1703–1855.

[5] M. P. Allen and D. J. Tildesley, *Computer Simulation of Liquids*, Oxford University PressOxford, 2nd edn, 2017.

[6] S. L. Mayo, B. D. Olafson and W. A. Goddard, *J. Phys. Chem.*, 1990, **94**, 8897–8909.

[7] A. K. Rappe, C. J. Casewit, K. S. Colwell, W. A. Goddard and W. M. Skiff, *J. Am. Chem. Soc.*, 1992, **114**, 10024–10035.

[8] M. N. Corella-Ochoa, J. B. Tapia, H. N. Rubin, V. Lillo, J. González-Cobos, J. L. Núñez-Rico, S. R. Balestra, N. Almora-Barrios, M. Lledós, A. Güell-Bara, J. Cabezas-Giménez, E. C. Escudero-Adán, A. Vidal-Ferran, S. Calero, M. Reynolds, C. Martí-Gastaldo and J. R. Galán-Mascarós, *J. Am. Chem. Soc.*, 2019, **141**, 14306–14316.

[9] A. García-Sánchez, C. O. Ania, J. B. Parra, D. Dubbeldam, T. J. H. Vlugt, R. Krishna and S. Calero, *J. Phys. Chem. C*, 2009, **113**, 8814–8820.

[10] A. Martín-Calvo, E. García-Pérez, A. García-Sánchez, R. Bueno-Pérez, S. Hamad and S. Calero, *Phys. Chem. Chem. Phys.*, 2011, **13**, 11165.

[11] T. D. Kühne, M. Iannuzzi, M. Del Ben, V. V. Rybkin, P. Seewald, F. Stein, T. Laino, R. Z. Khaliullin, O. Schütt, F. Schiffmann, D. Golze, J. Wilhelm, S. Chulkov, M. H. Bani-Hashemian, V. Weber, U. Borštnik, M. Taillefumier, A. S. Jakobovits, A. Lazzaro, H. Pabst, T. Müller, R. Schade, M. Guidon, S. Andermatt, N. Holmberg, G. K. Schenter, A. Hehn, A. Bussy, F. Belleflamme, G. Tabacchi, A. Glöß, M. Lass, I. Bethune, C. J. Mundy, C. Plessl, M. Watkins, J. VandeVondele, M. Krack and J. Hutter, *J. Chem. Phys.*, 2020, **152**, 194103.

[12] J. P. Perdew, K. Burke and M. Ernzerhof, *Phys. Rev. Lett.*, 1996, **77**, 3865–3868.

[13] S. Grimme, J. Antony, S. Ehrlich and H. Krieg, *J. Chem. Phys.*, 2010, **132**, 154104.

[14] S. Grimme, S. Ehrlich and L. Goerigk, *J. Comput. Chem.*, 2011, **32**, 1456–1465.

[15] J. VandeVondele and J. Hutter, *J. Chem. Phys.*, 2007, **127**, 114105.

[16] M. Krack, *Theor. Chem. Acc.*, 2005, **114**, 145–152.

[17] S. Goedecker, M. Teter and J. Hutter, *Phys. Rev. B*, 1996, **54**, 1703–1710.

[18] C. Hartwigsen, S. Goedecker and J. Hutter, *Phys. Rev. B*, 1998, **58**, 3641–3662.

[19] S. Sharma, S. R. G. Balestra, R. Baur, U. Agarwal, E. Zuidema, M. S. Rigutto, S. Calero, T. J. H. Vlugt and D. Dubbeldam, *Mol. Simul.*, 2023, **49**, 893–953.

[20] M. Polanyi, *Science*, 1963, **141**, 1010–1013.

[21] M. M. Dubinin, *Chem. Rev.*, 1960, **60**, 235–241.

[22] M. Polanyi, *Trans. Faraday Soc.*, 1932, **28**, 316–333.

[23] F. Stavarache, A. Luna-Triguero, S. Calero and J. M. Vicent-Luna, *Chemical Engineering Journal*, 2024, **496**, 153480.

[24] A. Hauer, *Chem. Ing. Tech.*, 2010, **82**, 1075–1080.

[25] Y. E. Gorbaty and G. V. Bondarenko, *J. Supercrit. Fluids*, 1998, **14**, 1–8.

[26] R. E. Walker and A. A. Westenberg, *J. Chem. Phys.*, 1958, **29**, 1139–1146.
